# Discovery of a Novel Benzothiophene Inhibitor of Penicillin-Binding Protein 2 with Potent Gram-Positive Activity

**DOI:** 10.3390/antibiotics15090826

**Published:** 2026-08-25

**Authors:** Monica A. Stefaniak, Vijay Singh Gondil, Jingdong Yang, Adil Omar, Allen G. Oliver, Ansley M. Nemeth, Roberta J. Melander, Mayland Chang, Paul M. Dunman, Christian Melander

**Affiliations:** 1 Department of Chemistry and Biochemistry, University of Notre Dame, Notre Dame, IN 46556, USA; mstefan2@nd.edu (M.A.S.); jiyang@chla.usc.edu (J.Y.); awumaier@nd.edu (A.O.); aoliver2@nd.edu (A.G.O.); anemeth2@nd.edu (A.M.N.); rmelande@nd.edu (R.J.M.); mchang@nd.edu (M.C.); 2 Department of Microbiology and Immunology, University of Rochester Medical Center, Rochester, NY 14642, USA; vijay_gondil@urmc.rochester.edu

**Keywords:** methicillin-resistant *Staphylococcus aureus*, antibacterial, benzothiophene, penicillin-binding proteins (PBPs), transglycosylase

## Abstract

**Background/Objectives**: The emergence of methicillin-resistant *Staphylococcus aureus* (MRSA) highlights the need for new antibacterial agents. Here, we report the identification and evaluation of a novel Gram-positive selective antibacterial: NDM-552. **Methods**: Antibacterial activity was determined by broth microdilution against a panel of Gram-positive and Gram-negative pathogens. Mechanistic studies included BOCILLIN-FL competition assays, microscale thermophoresis (MST), and antibiotic interaction with oxacillin and moenomycin. Additionally, time–kill analyses, frequency-of-resistance measurements, cytotoxicity assays, plasma stability studies, and a murine wound infection model were performed to characterize NDM-552. **Results**: NDM-552 exhibits activity against Gram-positive pathogens including *S. aureus*, *Enterococcus faecium*, and *Staphylococcus epidermidis*, with minimum inhibitory concentrations (MICs) of 1–2 µg/mL. NDM-552 displays no activity against Gram-negative bacteria. NDM-552 displays bacteriostatic activity against MRSA and additive interactions with oxacillin and moenomycin. The frequency of resistance in MRSA is low (2.39 × 10^−9^). BOCILLIN-FL competition assays and MST binding analysis identify the essential penicillin-binding protein 2 (PBP2) as the potential target of NDM-552. NDM-552 demonstrates acceptable cytotoxicity, favorable plasma stability, and reduces bacterial burden in a murine wound infection model. **Conclusions**: NDM-552 is a potent Gram-positive selective antibacterial agent that potentially targets PBP2-associated cell wall biosynthesis and exhibits in vivo efficacy against MRSA. Findings establish NDM-552 as a promising scaffold for the development of new antibacterial agents.

## 1. Introduction

Antibiotic resistance is a major and growing global health threat. *Staphylococcus aureus* is one of the six ESKAPE bacterial pathogens (*Enterococcus faecium*, *S. aureus*, *Klebsiella pneumoniae*, *Acinetobacter baumannii*, *Pseudomonas aeruginosa*, and *Enterobacter* species), a group that is a predominant cause of nosocomial infections and that is notorious for multidrug resistance [1]. *S. aureus* is responsible for a wide range of infections, including skin and soft tissue infections, pneumonia, and osteomyelitis, in both community and hospital settings [1,2,3]. Individuals at increased risk for *S. aureus* infections include health care workers, immunocompromised individuals, people with diabetes, children, older adults, and individuals using catheters, with these groups accounting for approximately 80% of cases [4]. Despite the urgent need for new therapeutics, reduced investment in antibiotic discovery by large pharmaceutical companies has slowed the development of novel agents [5].

ß-Lactam antibiotics, among the most effective treatments for *S. aureus* infections, act by inhibiting penicillin-binding proteins (PBPs) that catalyze the final steps of cell wall biosynthesis [6]. PBPs function primarily as transpeptidases responsible for crosslinking of peptidoglycan strands [7]. *S. aureus* expresses four native PBPs (PBP1, PBP2, PBP3, PBP4), of which PBP1 and PBP2 are essential for viability in laboratory culture conditions [6]. Historically, the methicillin-resistant *S. aureus* (MRSA) phenotype has been considered to be conferred by the acquisition of PBP2a, which has low affinity for most ß-lactam antibiotics [6]. More recently, a non-canonical PBP2a-independent MRSA pathway that involves PBP4 has been recognized to also confer high-level resistance to the ß-lactams. Vancomycin is the standard-of-care drug for many MRSA infections, but its effectiveness is restricted by nephrotoxicity and the emergence of resistant strains [8].

PBP2 is unique among *S. aureus* PBPs in that it possesses both transpeptidase and transglycosylase activity, the latter being essential for polymerizing glycan chains during peptidoglycan synthesis [9]. The essentiality of transglycosylase activity makes PBP2 an excellent antibacterial target. The natural product antibacterial moenomycin selectively inhibits the transglycosylation activity of PBP2 and validates PBP2 as a viable antibacterial target, although moenomycin’s poor pharmacological properties have limited its clinical use [10]. We previously reported benzothiophene-containing compounds, initially developed as *S. aureus* PBP4 inhibitors, with ß-lactam potentiation effects [11]. Subsequent analog synthesis led to the identification of compounds with enhanced oxacillin potentiation activity [12]. During this study, we discovered that one analog has potent standalone anti-staphylococcal activity. Here, we report the characterization of NDM-552 as a selective Gram-positive antibacterial agent, investigate its mechanism of action, with evidence supporting engagement with PBP2, and evaluate its in vivo activity in a murine wound infection model.

## 2. Results

### 2.1. Synthesis of NDM-552

**Scheme 1:**
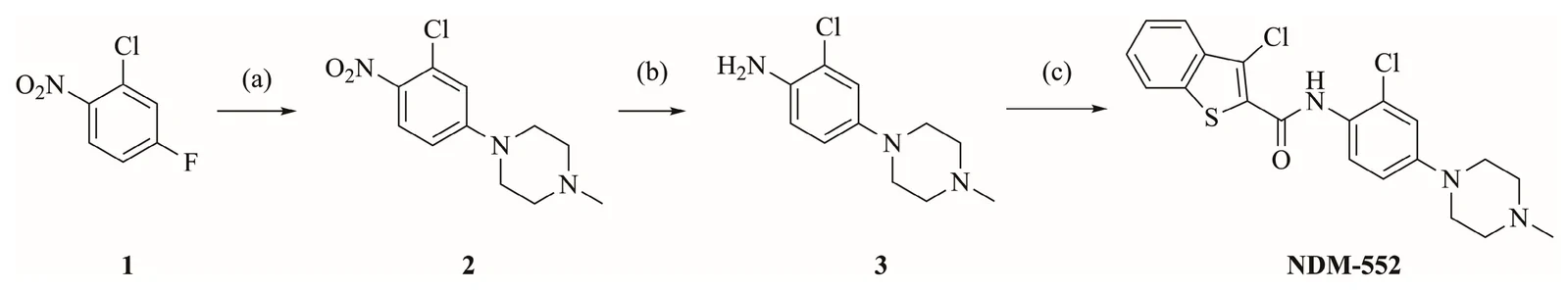
Synthetic route to NDM-552. (**a**) K_2_CO_3_, 1-methylpiperazine, DMF, 30 min. 95.3% yield; (**b**) SnCl_2_, HCl, EtOH, 80 °C, 1 h. 92.7% yield; (**c**) 3-chlorobenzothiophene-2-carbonyl chloride, TEA, DCM, rt, 12 h, 33.7% yield.

**Figure 1:**
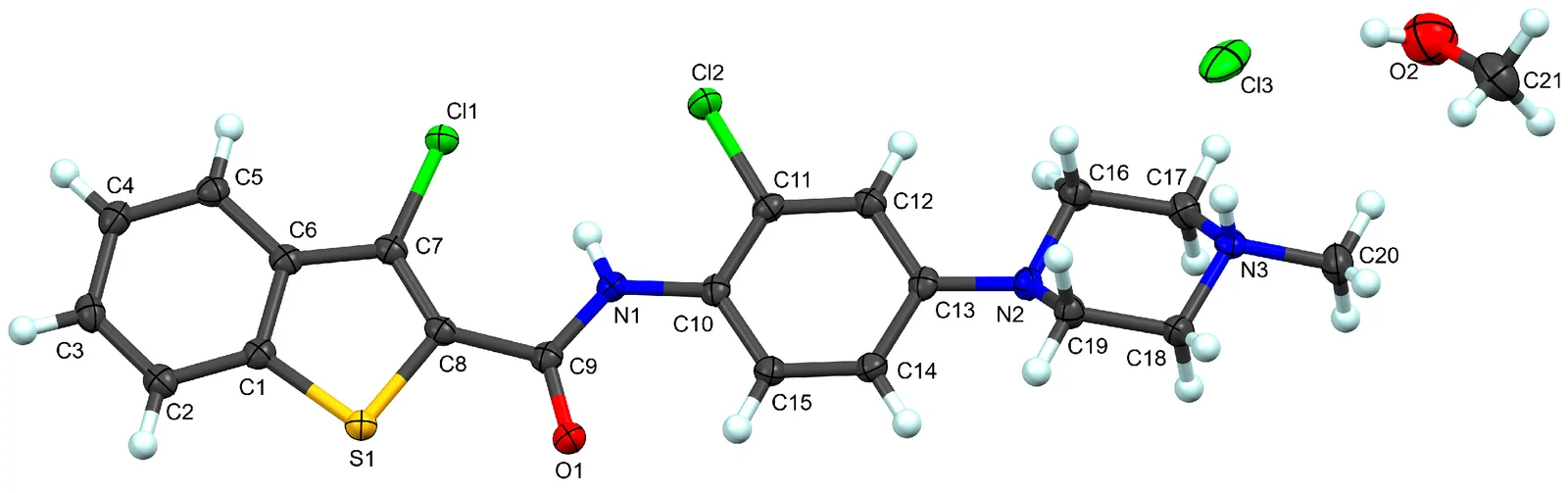
Crystal structure of the HCl salt of NDM-552. Black: carbon, White: hydrogen, Green: chlorine, Yellow: sulfur, Red: oxygen, Blue: nitrogen.

NDM-552 was synthesized via a nucleophilic aromatic substitution reaction between commercially available 2-chloro-4-fluoro-1-nitrobenzene **1** and 1-methylpiperazine to yield intermediate **2** (Scheme 1). A tin(II) chloride-mediated reduction of the nitro group afforded aniline **3**, which was subsequently acylated with 3-chlorobenzothiophene-2-carbonyl chloride to generate NDM-552. NDM-552 was treated with concentrated hydrochloric acid to yield the compound as the HCl salt. A crystal structure of the HCl salt of NDM-552 is provided in Figure 1.

### 2.2. Exploration of the Scope of Activity

First, we measured the standalone antibacterial activity of NDM-552 against a suite of Gram-positive bacteria, including MRSA, methicillin-susceptible *S. aureus* (MSSA), cystic fibrosis *S. aureus* (CFSA) clinical isolates, *E. faecium*, and *Staphylococcus epidermidis* (Table 1) using standard microdilution minimum inhibitory concentration (MIC) assays. Each strain returned a MIC of 1–2 µg/mL.

We then examined activity against Gram-negative bacteria, including *K. pneumoniae*, *A. baumannii*, and *P. aeruginosa*. The MICs of NDM-552 against *K. pneumoniae* ATCC BAA-2146, *A. baumannii* ATCC 19606, *A. baumannii* ATCC 17978, and PAO1 are all > 16 µg/mL (the highest concentration tested) (Table 2). To determine if the lack of Gram-negative antibacterial activity of NDM-552 is a result of its inability to penetrate the Gram-negative outer membrane, the MIC was determined against a lipooligosaccharide (LOS) deficient *A. baumannii* strain, AB 5075 LOS^−^, which was generated by growing AB 5075 on agar containing 10 µg/mL colistin as previously reported [13]. Upon observing growth (up to 48 h at 37 °C), a colony was cultured in CAMHB supplemented with 10 µg/mL colistin to ensure LOS deficiency. NDM-552 exhibits an MIC of >16 µg/mL against LOS-deficient AB 5075, potentially suggesting that the lack of activity against Gram-negative bacteria may be related to target specificity, not the ability of NDM-552 to penetrate the cell membrane in this specific organism. It should be noted that although LOS-deficient *A. baumannii* exhibit increased outer membrane permeability [14], this modification may not be sufficient to enable effective uptake of NDM-552. Given the moderately lipophilic nature of NDM-552 (clogP = 3.89, ChemDraw Professional, Version: 26.0.0.6599, Perkin Elmer, Waltham, MA, USA), additional permeability barriers, including limited passage across the cytoplasmic membrane or insufficient intracellular accumulation, may prevent the compound from reaching its cellular target. Another barrier for antibiotic activity in Gram-negative bacteria is the presence of efflux pumps [15]. To determine whether the lack of activity in Gram-negative cells is attributable to rapid efflux, the MIC of NDM-552 was determined against an *A. baumannii* strain lacking *adeIJK*, which encodes the resistance-nodulation-cell division (RND)-type efflux pump, AdeIJK [16]. The MIC of NDM-552 against AB 17978 ∆*adeIJK* is >16 µg/mL, suggesting that NDM-552 may not be a substrate of the AdeIJK efflux pump system, or NDM-552 is unable to enter the cell. To more empirically establish whether efflux systems impact NDM-552 susceptibility, two broad-spectrum efflux pump inhibitors (EPIs) were tested in combination with NDM-552: carbonyl cyanide *m*-chlorophenyl hydrazone (CCCP) and phenylalanine-arginine β-naphthylamide (PAβN). CCCP is known to reduce ATP production and increase membrane permeability in bacteria by interfering with the transmembrane electrochemical gradient and proton motive force [17]. PAβN is a Resistance-Nodulation-Division (RND)-pump family inhibitor that is also known to permeabilize the membrane of Gram-negative strains [18]. In combination with 60 µM CCCP or PAβN (30% of the standalone MIC of >200 µM) against AB 17978 or AB 5075, the MIC of NDM-552 remains at 16 µg/mL. In addition, the activity of NDM-552 against *K. pneumoniae* in the presence of polymyxin B nonapeptide hydrochloride (PMBN) was determined. PMBN is a cationic cyclic peptide derived by enzymatic processing from the naturally occurring peptide, polymyxin B [19,20]. PMBN permeabilizes Gram-negative outer membranes without killing bacterial cells [21]. At concentrations of PMBN up to 8 µg/mL, the MIC of NDM-552 in KP 2146 remains unchanged. Taken together, the data suggest that the lack of activity against the tested Gram-negative bacteria is not primarily attributable to reduced permeability or efflux in the evaluated strains and may instead reflect differences in target susceptibility. Additional studies across a broader range of Gram-negative species would be required to fully establish the determinants underlying the Gram-positive selectivity of NDM-552.

### 2.3. Time–Kill Curves Against MRSA ATCC 43300

**Figure 2:**
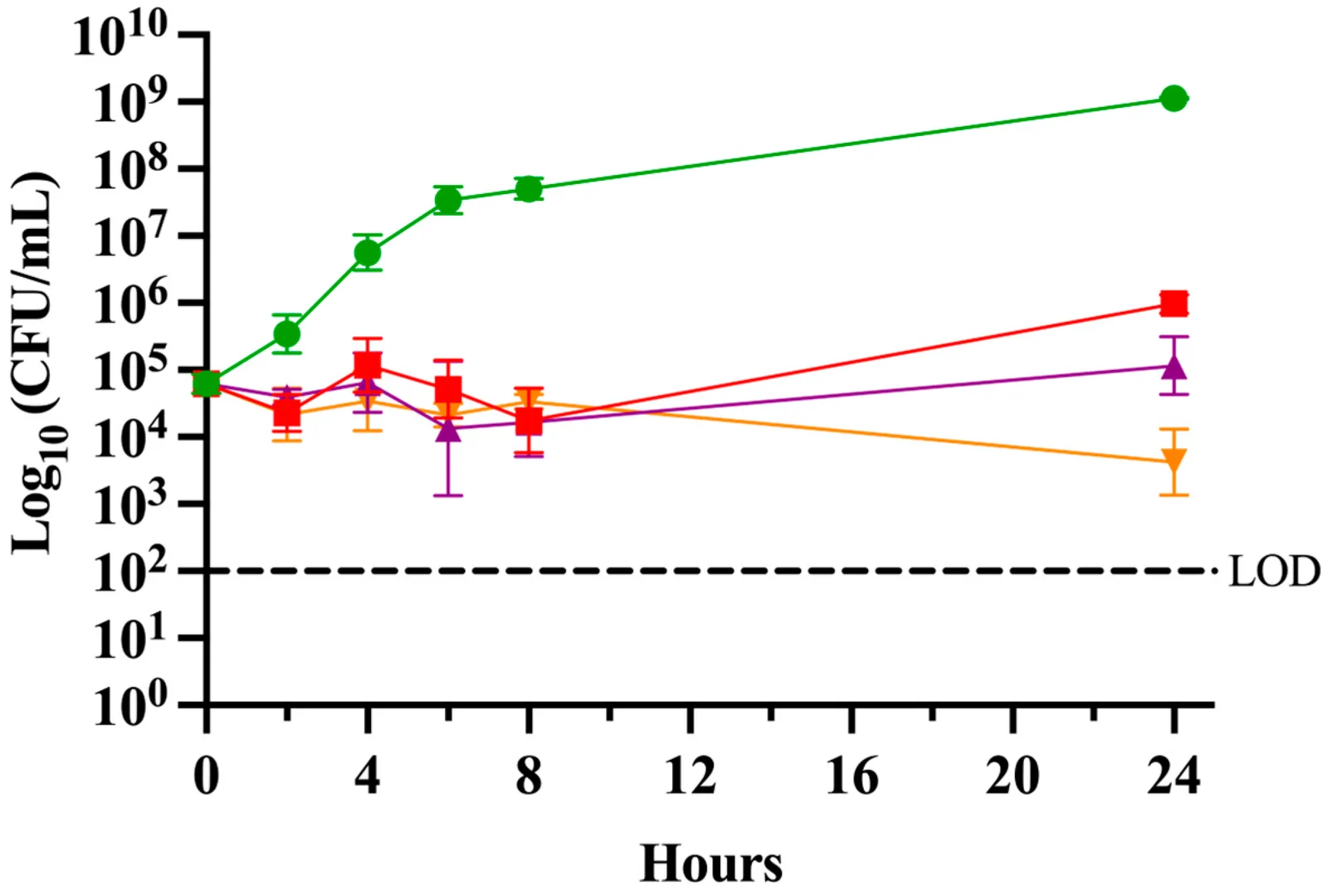
Time–kill curves for NDM-552. Green = untreated, red = 1× MIC, purple = 2× MIC, orange = 4× MIC. LOD = Limit of Detection. Data and SD from at least three replicates.

To establish whether the compound is likely to be bactericidal vs. bacteriostatic, a time–kill curve was constructed for NDM-552 using MRSA ATCC 43300 as a representative strain (Figure 2). NDM-552 was examined at its MIC (1 µg/mL), along with 2× and 4× this concentration. NDM-552 is bacteriostatic against MRSA 43300 at all concentrations tested [22].

### 2.4. Evaluation of NDM-552 MIC in the Presence of Fetal Bovine Serum (FBS) and Triton-X

The MIC of NDM-552 was determined in the presence of serum proteins to assess activity under physiologically relevant conditions. MICs were determined in a 9:1 mixture of cation-adjusted Mueller-Hinton Broth (CAMHB): fetal bovine serum (FBS) against MRSA ATCC BAA-1556 (Table S1). Under these conditions, the MIC increases 16-fold from 1 µg/mL to 16 µg/mL, consistent with compound sequestration by serum proteins and lipids, which reduces the freely available active concentration [23]. Direct measurement of serum protein binding and the fraction of unbound NDM-552 was not performed and represents a limitation of the current study. To evaluate whether the antibacterial activity of NDM-552 is influenced by compound aggregation, the MIC was also determined in the presence of 0.01% Triton X-100, a non-ionic detergent commonly used to disrupt aggregates [24]. The MIC of NDM-552 in the presence of 0.01% Triton X-100 is 1 µg/mL (Table S1), which suggests that the antibacterial activity of NDM-552 is not attributable to aggregation [25].

### 2.5. Determination of Synergy with Oxacillin

Because the benzothiophene family of compounds has been previously found to potentiate oxacillin toward MRSA [12], we performed a checkerboard assay with NDM-552 and oxacillin against MRSA ATCC BAA-1556 to learn whether the compound maintains adjuvant activity at a sub-inhibitory concentration. Fractional inhibitory concentration indices (FICI) were calculated to determine whether the combination is synergistic, additive, or antagonistic. The MIC of NDM-552 is reduced from 1 µg/mL to 0.5 µg/mL in combination with 8 µg/mL oxacillin, while oxacillin exhibits a decrease in MIC from 32 µg/mL to 8 µg/mL. The FICI is 0.75, indicating an additive interaction between NDM-552 and oxacillin (Table S2) [26].

### 2.6. Resistance to NDM-552

To determine the rate of resistance, MRSA ATCC BAA-1556 was serially passaged in the presence of 0.25×, 0.5×, 1×, 2×, and 4× the MIC of NDM-552 over ten days. MICs were recorded every second day for eight days (Table S3). Resistance in the bacterial population serially passaged with NDM-552 increased, with an MIC of 64 µg/mL on day two and >64 µg/mL on day four (highest concentration tested due to solubility). The evolved MRSA ATCC BAA-1556 strain with NDM-552 resistance will be referred to as MRSA 1556^R^.

### 2.7. Adjuvant Activity Against MRSA 1556^R^

We next evaluated whether NDM-552 exhibits adjuvant activity against the resistant mutant. The oxacillin MIC against this strain is 64 µg/mL, and NDM-552 lowers the oxacillin MIC to 0.125 µg/mL (512-fold) at 2 µg/mL, losing activity (oxacillin MIC 32 µg/mL) at 1 µg/mL (Table 3), implying that the compound displays two anti-staphylococcal mechanisms, acting as an antimicrobial agent and also as β-lactam adjuvant against strains that are resistant to NDM-552 antibacterial activity.

To determine if potentiation activity is specific to oxacillin, we tested NDM-552 (2 µg/mL) with other classes of antibiotics to which MRSA 1556^R^ is resistant. The MIC of azithromycin and bacitracin remain unchanged, while the tetracycline MIC decreases fourfold (Table S4).

### 2.8. Frequency of Resistance

To quantify the rate of resistance emergence, we performed spontaneous frequency of resistance (FoR) studies using MRSA ATCC BAA-1556 using standard Luria-Delbrück Fluctuation conditions [27,28]. NDM-552 displays a FoR of 2.39 × 10^−9^ at 8× the MIC (8 µg/mL). For reference, the frequency of resistance in MSSA to oxacillin is reported to be 10^−6^ at 2 µg/mL oxacillin [29].

### 2.9. BOCILLIN-FL Binding Assay

**Figure 3:**
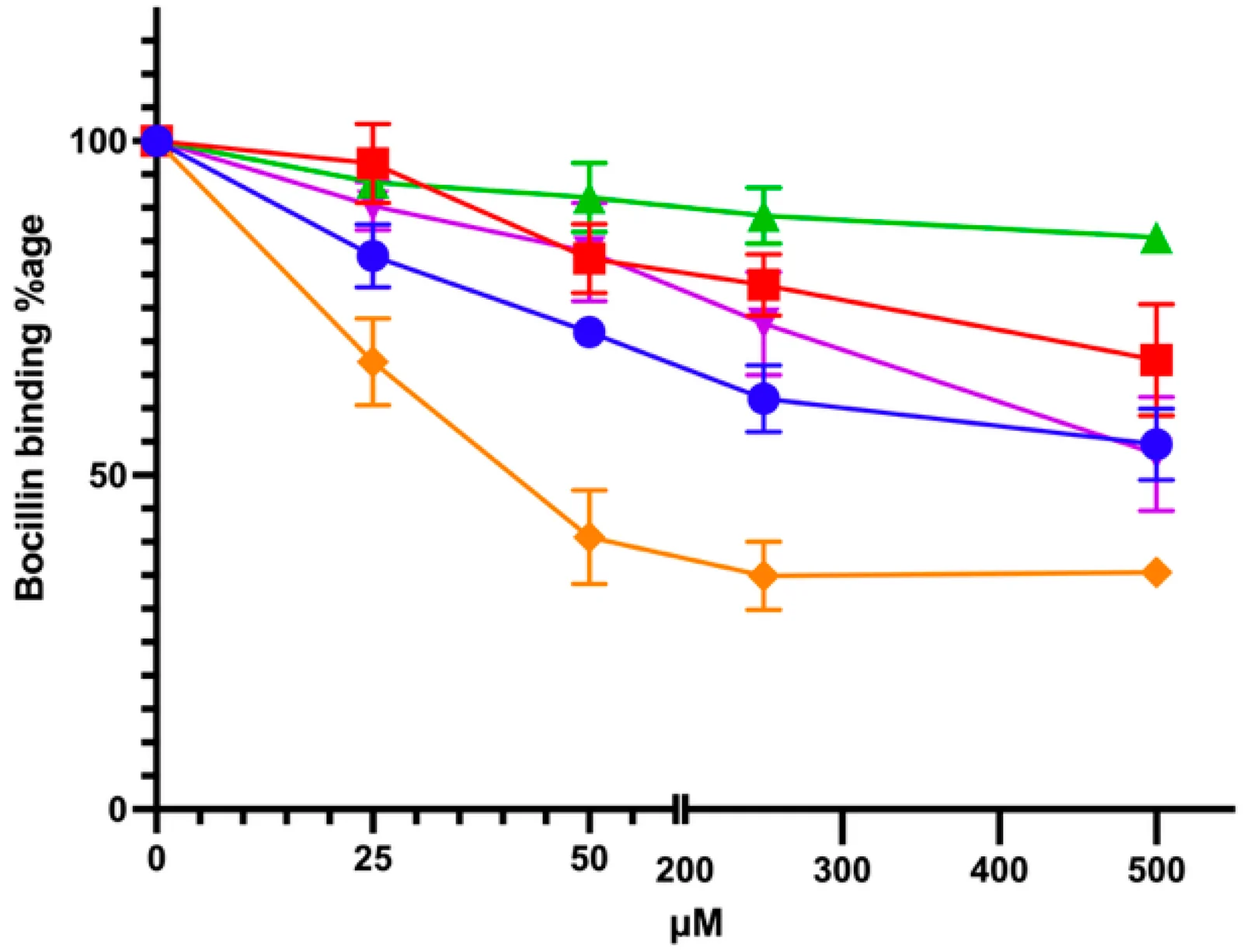
BOCILLIN-FL Binding assay of NDM-552. Blue: PBP1. Orange: PBP2. Red: PBP2a. Green: PBP3. Purple: PBP4.

To determine the relative binding of NDM-552 to PBP1, PBP2, PBP2a, PBP3, and PBP4, we performed a BOCILLIN-FL binding assay using recombinant PBPs from the MRSA strain COL and a BODIPY-labeled penicillin molecule. COL is a well-characterized MRSA clinical isolate that exhibits high-level β-lactam resistance. The percentage inhibition of BOCILLIN-FL binding in the presence of increasing concentrations of **NDM-552** is shown in Figure 3. IC_50_s are given in Table 4. NDM-552 more strongly inhibits BOCILLIN-FL binding to PBP2 (IC_50_ = 140.1 ± 73.8 µM) compared to other PBPs (IC_50_ = 886.1 ± 378.5 µM to 5393.9 ± 409.3 µM) (Table 4). A limitation of this assay is that BOCILLIN-FL irreversibly acylates the active site series of PBPs [30] and a well-established caveat of all β-lactam chromogenic PBP assay reporters is that they are irreversible PBP active-site modifiers and equilibrium greatly favors irreversible PBP acylation by the reporter. Thus, while PBP reporters accurately measure PBP active-site inhibition by covalent binders, they are known to significantly underestimate the activity of non-covalent active-site and/or allosteric binders [31,32].

### 2.10. Measurement of NDM-552 Binding to PBPs by Microscale Thermophoresis (MST)

We used microscale thermophoresis (MST) to quantify NDM-552 binding to purified PBPs from the MRSA strain COL. The K_Ds_ of NDM-552 for each protein are shown in Table 4. NDM-552 shows the strongest binding to PBP2 (K_D_ = 12.3 ± 8.5 µM) and PBP4 (K_D_ = 12.0 ± 9.2 µM) and moderate binding to PBP2a (K_D_ = 37.9 ± 20.4 µM).

### 2.11. Determination of Synergy with Moenomycin

To further investigate the mechanism of action of NDM-552, we performed a checkerboard assay in combination with moenomycin, a well-characterized inhibitor of the transglycosylase activity of PBP2 [33]. NDM-552 exhibits a reduction in MIC from 1 µg/mL to 0.25 µg/mL when combined with moenomycin against MRSA ATCC BAA-1556, while the moenomycin MIC decreases from 0.25 µg/mL to 0.125 µg/mL in combination with NDM-552 (Table S5). The FICI of 0.75 indicates an additive interaction between NDM-552 and moenomycin.

### 2.12. FDAA Incorporation Assays

**Figure 4:**
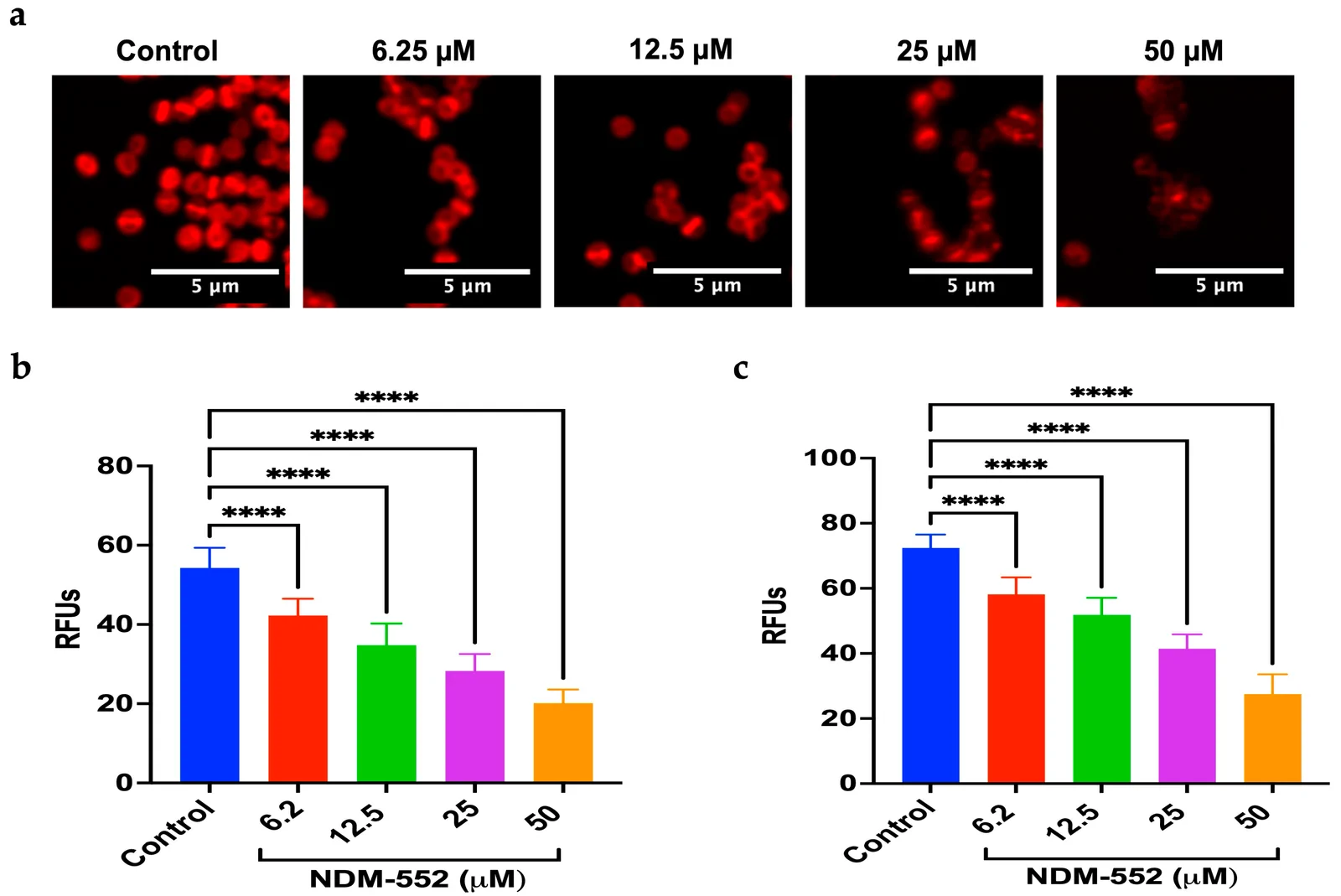
NDM-552 treatment reduced FDAA incorporation in the peptidoglycan of USA300 cells in a dose-dependent manner. Representative images (**a**) show the control and increasing concentrations of NDM-552 (6.25–50 μM), with corresponding decreases in fluorescence intensity. Quantification of RFUs for the peripheral cell wall (**b**) and the cell septal (**c**) is shown in the bar graphs. Data are mean ± SD; **** *p* < 0.0001.

Peptidoglycan synthesis proceeds through incorporation of lipid II precursors containing a terminal D-Ala-D-Ala motif, followed by PBP-catalyzed transglycosylation and transpeptidation. During transpeptidation, fluorescent D-amino acid (FDAA) probes can be incorporated into newly synthesized peptidoglycan, allowing visualization of sites of active cell wall synthesis [34]. In this study, we used TAMRA-D-Lys, a D-lysine probe conjugated to the red fluorescent tetramethyl rhodamine (TAMRA) fluorophore, to monitor peptidoglycan synthesis. To evaluate the effect of NDM-552 on peptidoglycan crosslinking, *S. aureus* USA300 cells were grown in the presence of FDAA and increasing concentrations of NDM-552. As shown in Figure 4a, in the absence of NDM-552 cells exhibit 54.25 ± 5.08 RFUs/cell, which dose-dependently decreases upon treatment with NDM-552: 42.26 ± 4.21 RFUs/cell at 6.2 µM NDM-552, 37.74 ± 5.49 RFUs/cell at 12.5 µM, 28.25 ± 4.29 RFUs/cell at 25 µM, and 20.15 ± 3.44 RFUs/cell at 100 µM NDM-552. As PBP2 is recruited to the division site to form the cell septum [35], the effects of NDM-552 on FDAA incorporation at the cell septum were investigated. Similar to peripheral FDAA incorporation into peptidoglycan, the cell septum also showed limited FDAA incorporation upon treatment with NDM-552. Untreated cells showed 72.43 ± 4.08 RFUs/cell septum, which decreased upon treatment with NDM-552—58.18 ± 5.22 RFUs/cell septum at 6.25 µM, 51.82 ± 5.26 RFUs/cell septum at 12.5 µM, 41.41 ± 4.45 RFUs/cell septum at 25 µM, and 27.50 ± 6.06 RFUs/cell septum at 100 µM NDM-552 (Figure 4b).

### 2.13. Cytotoxicity and Therapeutic Index

Before examining the in vivo activity of NDM-552, the therapeutic index (TI) was established. The effect of NDM-552 on the viability of the hepatocellular carcinoma cell line, HepG2, was determined (Table 5). The TI for conventional antibiotic development is defined as (mammalian cell CC_50_)/(antibiotic MIC), with a TI ≥ 50 desirable for further development. The CC_50_ of NDM-552 against HepG2 cells is 141.8 µM; with MICs between 1 and 2 µg/mL (2.19–4.38 µM), this gives a TI ranging from 32.4 to 64.8.

### 2.14. Preliminary Pharmacokinetic Data

NDM-552 was assessed for metabolic stability in mouse S9 fractions and for stability in mouse plasma. After four hours, NDM-552 showed high plasma stability (88.2%) and high internal clearance (224 mL/min/kg). Finally, the microsomal half-life of NDM-552 is 5.93 min (Table 5).

### 2.15. Murine Wound Model

**Figure 5:**
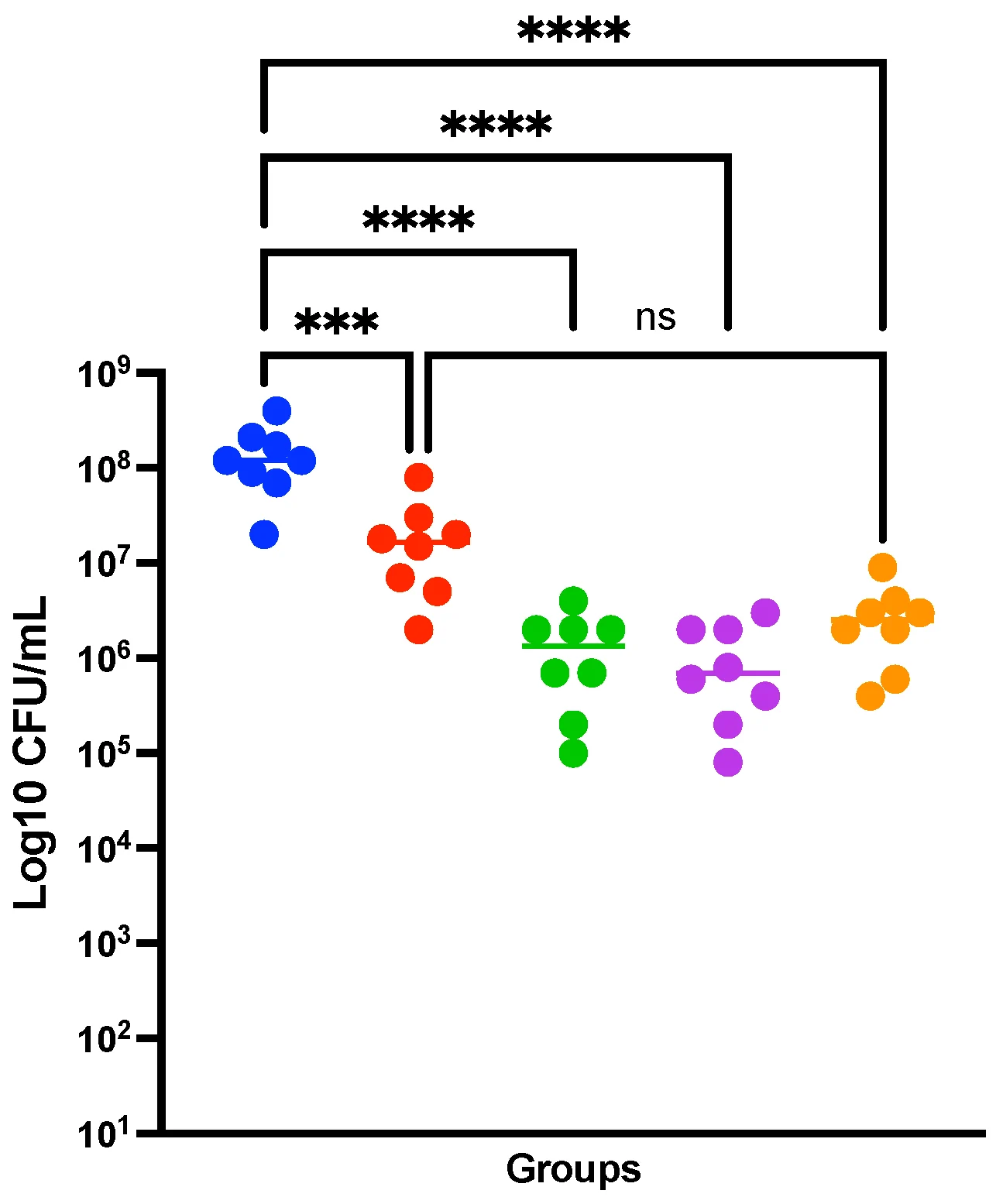
Murine wound infection model at 1×, 2×, and 5× MIC of NDM-552. Blue: USA300. Red: 1 µg/mL NDM-552. Green: 2 µg/mL NDM-552. Purple: 5 µg/mL NDM-552. Orange: 0.25 µg/mL ceftobiprole. *** indicates *p* < 0.001. **** indicates *p* < 0.0001. “ns” indicates “not significant”.

The in vivo activity of NDM-552 was evaluated in a murine wound infection model. Female BALB/c mice bearing a 6 mm full-thickness dermal wound were infected with 10^5^ CFU MRSA USA300. Following infection, mice received NDM-552 treatment every six hours for three days. At the end of the study, infected wound tissue was excised and the bacterial burden was quantified. Administration of NDM-552 at 1× MIC produced a significant reduction in bacterial counts, while treatment at 2× and 5× MIC resulted in a dose-dependent reduction, with the highest dose producing a significant decrease relative to the untreated group (Figure 5). The antibacterial activity of NDM-552 at 5× MIC was comparable to that observed for ceftobiprole, indicating that NDM-552 is effective at reducing MRSA burden in vivo.

## 3. Discussion

Increasing incidence of multidrug-resistant *S. aureus* continues to create an urgent need for antibacterial agents that operate through mechanisms distinct from those of existing antibiotics. In this study, we identify NDM-552 as a potent benzothiophene-derived antibacterial agent with selective activity against Gram-positive pathogens and uncover evidence supporting PBP2 as its potential molecular target. Collectively, the data establish NDM-552 as a promising lead for further antibacterial development that expands the chemical space of non-β-lactam PBP inhibitors.

NDM-552 demonstrates consistent activity against clinically relevant MRSA, MSSA, cystic fibrosis *S. aureus* clinical isolates, *E. faecium*, and *S. epidermidis* strains, with MICs ranging from 1 to 2 µg/mL, but limited activity against permeability-enhanced and efflux-deficient *A. baumannii* strains. These experiments suggest that reduced outer membrane permeability and the AdeIJK efflux system are unlikely to be the sole determinants of the observed selectivity. In addition, the compound may still experience limited passage across the cytoplasmic membrane or insufficient intracellular accumulation due to its lipophilic character. Structural differences between *S. aureus* PBP2 and *A. baumannii* PBP1a may contribute to the lack of activity against Gram-negative bacteria. Both enzymes contain conserved transglycosylase and transpeptidase domains required for peptidoglycan biosynthesis *S. aureus* PBP2 lacks an outer-membrane regulatory partner and relies on structural flexibility for activation. The “Jaw” subdomain functions as a hinge that undergoes conformational rearrangement upon interaction with the membrane, enabling regulation of transglycosylase activity [36]. In contrast, *A. baumannii* PBP1a possesses a more rigid, β-rich linker domain connecting the transglycosylase and transpeptidase domains and relies on interaction with the outer-membrane lipoprotein LpoA for activation [37]. Binding of LpoA to regulatory regions of PBP1a induces conformational changes within the linker domain that allosterically open the transglycosylase active site to facilitate Lipid II polymerization [38]. These differences potentially reduce recognition of compounds optimized for *S. aureus* PBP2.

Time–kill studies demonstrate that NDM-552 exhibits a bacteriostatic phenotype against MRSA agent inhibiting bacterial proliferation (<3-log reduction). The FoR observed for NDM-552 is 2.39 × 10^−9^, which is approximately three orders of magnitude lower than that reported for oxacillin in MSSA, indicating a lower likelihood of spontaneous resistance emergence [29]. Despite a low FoR, rapid resistance development observed in the creation of the resistant strain (Table S4) suggests that resistance-conferring mutations can be strongly selected for under sustained drug pressure.

Oxacillin-specific adjuvant activity against an NDM-552-resistant strain (MRSA 1556^R^) may be a result of the molecular target of NDM-552 having an influence on oxacillin resistance. Given that oxacillin resistance in MRSA is primarily mediated by the low-affinity transpeptidase PBP2a, which cooperates with the transglycosylase activity of native PBP2 [39], the oxacillin-specific adjuvant activity of NDM-552 is consistent with inhibition of PBP2. Disruption of PBP2-mediated glycan chain polymerization is proposed to impair PBP2a’s ability to compensate for β-lactam inhibition of native PBPs, thereby restoring oxacillin susceptibility [40,41,42].

Multiple orthogonal experiments support interaction of NDM-552 with PBP2, although they do not establish PBP2 as the sole or definitive cellular target. BOCILLIN-FL competition assays reveal preferential inhibition of PBP2 labeling relative to other PBPs, while MST measurements detect binding to purified PBP2 and PBP4. Although measurable binding to PBP4 was observed, it is unlikely that the standalone antibacterial activity of NDM-552 is exclusively due to PBP4 inhibition because PBP4 is not essential for *S. aureus* viability [43]. The interaction of NDM-552 with PBP2 suggests that PBP2 inhibition may contribute to the antibacterial activity; however, additional studies are required to establish the relative contribution of PBP2 inhibition to the observed phenotype.

The additive interaction observed between NDM-552 and moenomycin, a well-characterized transglycosylase inhibitor, is consistent with both compounds affecting the same essential pathway [33]. Although additive interactions cannot definitively establish a shared binding site or mechanism, the absence of synergy suggests a similar mechanism of action. Additionally, the additive interaction between NDM-552 and moenomycin suggests that they do not compete for the same binding site and supports a model in which both compounds impair the same essential enzymatic function through non-identical binding modes, further suggesting PBP2 as a putative functional target of NDM-552. FDAA incorporation assays establish that NDM-552 inhibits PBP2-mediated D-Ala incorporation in the periphery and cell septum of *S. aureus* cells.

In a murine wound model, NDM-552 (1 µg/mL) effects a statistically significant decrease in bacterial burden (Figure 5). In addition, a dose-dependent effect of NDM-552 was observed, with increasing efficacy observed as the concentration increased from 1 to 5 µg/mL. Treatment with 2 µg/mL and 5 µg/mL resulted in a 2-log reduction in the bacterial burden, comparable to the 2-log reduction achieved with 0.25 µg/mL BPR. Metabolic stability studies reveal rapid clearance of NDM-552 in mouse S9 fractions, suggesting that further medicinal chemistry optimization is required to improve pharmacokinetic properties. Nevertheless, the compound maintains sufficient exposure to demonstrate topical efficacy in a wound infection model.

Few non-β-lactam PBP-targeting compounds have been reported with both quantitative biochemical inhibition values and corresponding MRSA MIC data. The oxadiazole PBP2a inhibitors reported by Mobashery represent one such example, exhibiting MRSA MIC values of 1–2 µg/mL and PBP2a IC_50_ values of 4–8 µg/mL [44]. Although these compounds target PBP2a, they provide a useful benchmark for evaluating the relationship between PBP inhibition and antibacterial activity among non-β-lactam PBP-targeting scaffolds.

Overall, NDM-552 represents a promising antibacterial lead with a distinct mechanism of action from ß-lactam antibiotics. The combination of potent anti-MRSA activity, evidence for PBP2 engagement, low spontaneous resistance frequency, and in vivo efficacy supports continued optimization of this scaffold. Future studies will focus on defining the precise binding site of NDM-552, determining whether transglycosylase inhibition is the primary mechanism of action, and improving pharmacokinetic properties to enable systemic or topical administration.

## 4. Materials and Methods

### 4.1. Broth Microdilution Method for Minimum Inhibitory Concentration (MIC) Determination

Bacteria were cultured for six hours in CAMHB, then subcultured to 5 × 10^5^ CFU/mL in CAMHB or MHB (CRB, COL). Subcultures were aliquoted (1 mL) into culture tubes and then dosed with compound from an 8 mg/mL stock in DMSO to the desired concentration. Samples were aliquoted (200 µL) into the first row of wells in the 96-well microtiter plate. Rows 2–11 were filled (100 µL) with bacterial subculture. From row 1, 100 µL was transferred to row two and mixed 5–8 times before being transferred to row 3. This procedure was used to serially dilute the remaining rows through 10. The final row was filled with 100 µL of sterile media. The microtiter plate was sealed with Press ‘n Seal film (Glad, Oakland, CA, USA) and incubated at 37 °C for 16–20 h. Following incubation, bacterial growth was quantified by measuring the optical density at 600 nm (OD_600_) using a BioTek Synergy HTX Multimode plate reader (Santa Clara, CA, USA). The minimum inhibitory concentration (MIC) was defined as the lowest compound concentration that reduced growth to less than 10% of the positive control. A control experiment with 0.4% DMSO showed no growth inhibition in representative MRSA strains (MRSA 1556, MRSA 43300, and MRSA AH-1263).

### 4.2. Broth Microdilution Method for Potentiation of Antibiotics in Combination with NDM-552

Bacteria were cultured for six hours in CAMHB, then subcultured to 5 × 10^5^ CFU/mL in CAMHB or MHB (COL, CRB). Subcultures were aliquoted (4 mL) into culture tubes and then dosed with compound from an 8 mg/mL stock in DMSO to the desired concentration. The highest concentration tested in this assay (5 µg/mL) does not exceed 0.0625% DMSO. To a secondary culture tube, 1 mL was aliquoted and dosed with the antibiotic at the highest concentration to be tested. Bacteria treated with the antibiotic alone served as the control. Samples from the secondary culture tubes were then aliquoted (200 µL) into the first row of wells of a 96-well microtiter plate. Rows 2–11 were filled (100 μL) with the remaining 4 mL of bacterial subculture containing the adjuvant. Row 12 was filled (100 μL) with sterile media. From row 1, 100 μL was transferred to row two and mixed 5–8 times before being transferred to row 3. This procedure was used to serially dilute the rest of the rows through 10. The microtiter plate was sealed with Press ‘n Seal film and incubated at 37 °C for 16–20 h. Following incubation, bacterial growth was quantified by measuring the optical density at 600 nm (OD_600_) using a BioTek Synergy HTX Multimode plate reader. The minimum inhibitory concentration (MIC) was defined as the lowest compound concentration that reduced growth to less than 10% of the positive control. A control experiment with 0.4% DMSO showed no growth inhibition of MRSA 1556, MRSA 43300, and MRSA AH-1263.

### 4.3. Preparation of LOS-Deficient A. baumannii Mutants

LOS-deficient *A. baumannii* mutants were obtained following a previously published procedure. Selective agar plates containing 10 μg/mL colistin were prepared before mutant isolation. *A. baumannii* strain 5075 was grown in CAMHB for 8 h, and a 125 μL aliquot of the culture was plated onto the selective medium. The inoculum was evenly distributed across the agar surface using sterile glass beads (MilliporeSigma, Burlington, MA, USA). Plates were incubated at 37 °C for up to 48 h, after which emerging colonies were transferred into CAMHB supplemented with 10 μg/mL colistin for propagation. These cultures were subsequently used for the assays described above.

### 4.4. BOCILLIN-FL Binding Assay

The binding of NDM-552 to *S. aureus* PBPs (PBP1, PBP2, PBP2A, PBP3, and PBP4) was assessed using the gel-based BOCILLIN-FL assays, as described earlier [11,45]. Briefly, 1 μM of each purified PBP was incubated with increasing concentrations (0, 25, 50, 250, and 500 μM) of NDM-552 in the 50 mM phosphate buffer (pH 7.4) at 37 °C for 30 min. After 30 min incubation, 40 µM of BOCILLIN-FL (penicillin V conjugated to BODIPY FL dye; Fisher Scientific, Hampton, NH, USA) was added to each reaction and incubated for an additional 30 min at 37 °C. Reactions were loaded and resolved on a 12.5% SDS-PAGE gel, then stained with Biosafe-Coomassie dye. Fluorescence of BOCILLIN-FL and protein bands was detected using the BioRad Gel Doc EZ Imaging system (BioRad, Hercules, CA, USA,). The intensity of BOCILLIN and protein bands was analyzed using ImageJ software (NIH) (Version 1.54t), and fluorescence was normalized to the protein intensity in each reaction. The data were presented as BOCILLIN binding inhibition, comparing the normalized fluorescence signal from the tested compound (PBP+ BOCILLIN-FL+NDM-552) with that of the untreated control samples (PBP+ BOCILLIN-FL).

### 4.5. Determination of Binding Affinity of NDM-552 to PBPs by Microscale Thermophoresis (MST)

Purified PBPs were fluorescently labeled with RED-NHS 2nd Generation dye (NanoTemper Technologies, Munich, Germany) according to the manufacturer’s instructions. Briefly, 100 μL of a 10 μM PBP solution was exchanged into labeling buffer (130 mM NaHCO_3_, 50 mM NaCl, pH 8.2–8.3) using the provided buffer exchange column (NanoTemper Technologies) by centrifugation at 1500× *g* for 2 min. The buffer-exchanged protein (90 μL) was then incubated with 10 μL of a 300 μM RED-NHS dye solution prepared in DMSO for 30 min at room temperature in the dark. Following labeling, excess unreacted dye was removed using a PD-10 desalting column (NanoTemper Technologies) equilibrated with 1× PBS containing 0.05% Tween-20. The concentration of the labeled PBP was calculated according to the manufacturer’s labeling protocol.

Binding interactions between fluorescently labeled PBPs and test compounds were initially evaluated using microscale thermophoresis (MST) on a Monolith NT.115 Pico instrument (NanoTemper Technologies, Munich, Germany). To prepare 800 µM NDM-552 in PBS, NDM-552 was initially prepared as a 100 mM stock solution in 100% DMSO and diluted to a 20 mM working stock solution in DMSO. The 20 mM DMSO stock was further diluted by transferring 2 μL of compound solution into 48 μL assay buffer (1× PBS containing 0.05% Tween-20), yielding an 800 μM compound solution in PBS containing 4% DMSO. Following serial dilution, 10 μL of the 800 µM solution was mixed with 10 μL of labeled target protein in PBS, resulting in a final assay concentration of 400 µM and a final DMSO concentration of 2%, which is acceptable according to Monolith NT.115 manufacturer’s instructions. An 80 µM stock solution is also used and prepared by diluting an 800 µM stock (containing 4% DMSO) 10-fold using PBS (final DMSO concentration 0.4%). Fluorescently labeled PBP was diluted to 10 nM in assay buffer. Prior to MST analysis, all solutions were centrifuged at 14,000× *g* for 5 min to remove any insoluble material, and the resulting supernatants were transferred to fresh microcentrifuge tubes. Equal volumes (25 μL) of labeled PBP and 80 μM compound solution were mixed and loaded into four MST capillaries. Control capillaries containing labeled PBP in assay buffer alone were prepared in parallel. Binding was assessed by comparing the thermophoretic responses of compound-treated samples with those of the corresponding controls. MST data were analyzed using MO.Affinity Analysis software (Version 2.3, NanoTemper Technologies, Munich, Germany).

For compounds exhibiting measurable binding interactions, MST was subsequently used to determine dissociation constants (K_D_). Compound solutions were prepared at an initial concentration of 800 μM in assay buffer containing 8% DMSO and serially diluted two-fold in assay buffer (1× PBS containing 0.05% Tween-20) to generate a concentration range of up to 16 points. Fluorescently labeled PBP was diluted to 10 nM, and 10 μL of protein solution was mixed with 10 μL of each compound dilution. The resulting samples were loaded into 16 MST capillaries and analyzed using the Monolith NT.115 Pico instrument. Thermophoretic traces were processed using MO.Affinity Analysis software to calculate K_D_ values. All binding measurements were performed in triplicate.

### 4.6. Evolution of NDM-552 Resistance in MRSA

Methicillin-resistant *S. aureus* ATCC BAA-1556 was cultured overnight in CAMHB and diluted into fresh CAMHB to achieve a starting inoculum of approximately 5 × 10^5^ CFU/mL in a total volume of 6 mL. The bacterial suspension was divided into five 1 mL aliquots, and NDM-552 was added from a DMSO stock solution to achieve concentrations corresponding to 0.25×, 0.5×, 1×, 2×, and 4× the established MIC. DMSO stock solutions were added to 1 mL aliquots such that the DMSO concentration did not exceed 0.2%. Cultures were incubated overnight at 37 °C with shaking. Following incubation, bacterial growth was assessed by visual turbidity, and the culture containing the highest concentration of NDM-552 that permitted visible growth was selected for serial passage. This culture was diluted back to approximately 5 × 10^5^ CFU/mL in fresh CAMHB containing the corresponding NDM-552 concentration and incubated under the same conditions. Serial passaging was continued for eight consecutive days, with MIC values determined by broth microdilution every 48 has described above.

### 4.7. Time–Kill Curves

Methicillin-resistant *S. aureus* ATCC 43300 was grown overnight in CAMHB and diluted into fresh CAMHB to obtain a starting inoculum of approximately 8 × 10^5^ CFU/mL. The resulting cultures were distributed into 3 mL aliquots in separate culture tubes and treated with NDM-552 at concentrations corresponding to 1×, 2×, or 4× the MIC. An untreated culture was included as a growth control. All samples were incubated at 37 °C under static conditions. At designated time points (2, 4, 6, 8, and 24 h), 100 μL samples were collected from each culture and subjected to serial ten-fold dilution in PBS (up to a 10^7^-fold dilution). Aliquots (100 μL) from the dilution series were plated onto tryptic soy agar (TSA) plates and incubated overnight at 37 °C. Following incubation, colony-forming units were quantified using a SphereFlash colony counter (NEUTEC Group Inc., Farmingdale, NY, USA). Time–kill curves were generated and analyzed using GraphPad Prism (Version 11.0.2).

### 4.8. Frequency of Resistance–Fluctuation Analysis

Methicillin-resistant *S. aureus* ATCC 1556 was cultured overnight in CAMHB at 37 °C, shaking at 200 rpm. The overnight culture was diluted 100-fold in 1X PBS and subsequently 1000-fold in fresh CAMHB to exclude any pre-existing mutants. The diluted culture was incubated for 24 h at 37 °C with shaking at 200 rpm. After 24 h, the culture was diluted to an OD600 of 0.00009 in 5 mL and 50 mL of fresh CAMHB in the absence or presence of NDM-552, respectively. Compound NDM-552 was added to the 50 mL subculture at 8 µg/mL (8× MIC) from a DMSO stock solution to be used as the master inoculum for the detection of mutants. To a deep-well 96-well plate, 1 mL aliquots were transferred to 40 individual wells. The plate was covered with Press ‘n Seal and incubated stationary at 37 °C for 16 h. The 5 mL subculture was not dosed and used for colony-forming unit (CFU) enumeration of the untreated inoculum. For CFU determination, the untreated subculture was serially diluted in 1× PBS, plated on tryptic soy agar (TSA), and incubated at 37 °C for 16 h. After incubation, the number of clear wells was determined, and CFU were enumerated. The mutation rate (µ) was estimated using the *Po* method [27].

### 4.9. HepG2 Cell Toxicity

A mammalian cell cytotoxicity test of NDM-552 was conducted using HepG2 human liver cancer cells (ATCC HB-8065, Manassas, VA, USA) in accordance with International Organization for Standardization guidelines. Briefly, HepG2 cells were grown in Dulbecco’s modified Eagle medium (Thermo Fisher Scientific, Waltham, MA, USA) containing 1% penicillin and streptomycin and 10% heat-inactivated fetal bovine serum. Cells were cultivated at 37 °C with 5% CO_2_ until 80–90% confluency. Cells were resuspended in 0.25% trypsin and washed in DMEM (Corning, Corning, NY, USA) and seeded in 96-well plates (5000 cells per well). Overnight, plates were incubated at 37 °C with 5% CO_2_. The following day, 100 µL of fresh media were supplemented with 2% (*v*/*v*) NDM-552 at final concentrations ranging from 0 to 100 μg/mL; DMSO and 125 μg/mL mitomycin C served as negative (solvent) and positive controls, respectively. The cells were then washed with 1x phosphate-buffered saline (PBS), and the media from each well were replaced with fresh DMEM and 10 μL of 12 mM (3-(4,5-dimethylthiazol-2-yl)-2,5-diphenyltetrazolium bromide (MTT) reagent (Cyquant Cell Viability kit; Invitrogen, Carlsbad, CA, USA). The plates were then incubated for a further 24 h at 37 °C. After incubation, 85 µL of media was removed, and 50 µL of DMSO was added to each well. The plate was further incubated at 37 °C for 4 h, and OD was recorded at 540 nm after incubation. Cell viability was expressed as relative viability for NDM-552-treated cells compared with DMSO-treated cells.

### 4.10. Metabolic Stability Assessment

Metabolic stability assessment was conducted using CD-1 mouse liver S9 fractions. The liver S9 fractions were thawed gradually on ice and maintained on ice throughout the preparation process. Then, 0.4 µL 1 mM test compounds (1 µM final concentration) were added to 372 µL reaction mixtures containing 1 mg/mL mouse S9 fraction in 10 mM PBS (pH 7.4), and pre-incubated at 37 °C for 5 min before being initiated by the addition of the cofactors, including 20 mM NADPH (20 µL) and 250 mM UDPGA (8 µL). The mixtures were incubated at 37 °C for up to 60 min with gentle shaking. At time points (0, 5, 15, 30, 45, and 60 min), 50 µL aliquots were withdrawn and quenched with 150 µL of 90% acetonitrile containing the internal standard (100 nM). Quenched samples were centrifuged at 15,000× *g* for 5 min, and the resulting supernatants were collected for LC–MS analysis.

A 5 μL sample aliquot was subjected to LC-MS/MS analysis using an ACQUITY UPLC I-Class PLUS System coupled to a Waters Xevo TQD mass spectrometer (Waters, Milford, MA, USA). Data were acquired in multiple reaction monitoring (MRM) mode using electrospray ionization (ESI). Chromatographic separation was achieved on an Acclaim RSLC 120 C18 column (2.2 μm particle size, 120 Å pore size, 2.1 × 100 mm) with a mobile phase consisting of water (solvent A) and acetonitrile (solvent B), each supplemented with 0.1% formic acid. The separation was performed using a gradient program beginning at 5% solvent B for 1 min, followed by a linear increase to 100% B over 2 min. The column was maintained at 100% B for 1.5 min before re-equilibration to the starting conditions. The flow rate was maintained at 0.4 mL/min throughout the analysis. The transitions monitored were *m*/*z* 420 → 58, *m*/*z*, 420 → 195 for NDM-552 and *m*/*z* 380 → 127, m/z 380 → 155 for NDM-365 (internal standard) [12]. The compound half-life (t_1/2_) was derived from plots of the natural log of the percent of remaining compound over time to determine the gradient of the line. The following equations are used to analyze data from this assay:

Elimination rate constant (k) = −(gradient)

Half-life (t_1/2_) (min) = ln2/k

Clint, app = (0.693/t_1/2_) × (incubation volume/mg microsomal protein) × (45 mg microsomal protein/g of liver) × (42.5 g of liver/kg body weight)

### 4.11. Mouse Plasma Stability Assessment

A 400 µL mouse plasma (CD-1 mouse) spiked with 0.4 µL 1 mM test article (final conc 1 µM) was mixed after vortexing for 1 min and incubated at 37 °C for up to 2 h. Then, 50 µL aliquots of plasma were removed at chosen time points (0, 5, 15, 30, 60, 120 min), 50 µL distilled water was added, and proteins were precipitated by the addition of 200 µL 90% ACN containing the internal standard (100 nM). Centrifuged at 15,000× *g*, 4 °C for 10 min. The supernatant was analyzed using LC-MS. A 5-μL aliquot was analyzed using an ACQUITY UPLC I-Class PLUS System and a Waters Xevo TQD (Waters, Milford, MA, USA) mass spectrometer in the MRM scan mode with ESI. The mobile phase consisted of water (A) and acetonitrile (B), both containing 0.1% formic acid. The chromatography was performed on an Acclaim RSLC 120 C18 column (2.2 µm, 120 Å, 2.1 ID × 100 mm) using a gradient elution method (5% B from 0 to 1 min, then to 100% B over 2min, and held at 100% B for an additional 1.5 min before returning to 5% B) at a flow of 0.4 mL/min. The transitions monitored were *m*/*z* 420 → 58, *m*/*z* 420 → 195 for NDM-552 and *m*/*z* 380 → 127, *m*/*z* 380 → 155 for NDM-365 (internal standard) [12].

### 4.12. Murine Wound Model

Four- to six-week-old female BALB/c mice (Charles River Laboratories, Wilmington, MA, USA) were anesthetized by intraperitoneal administration of ketamine (100 mg/kg; Hikma Pharmaceuticals, Berkley Heights, NJ, USA) and xylazine (10 mg/kg; Akorn Inc., Lake Forest, IL, USA). Pain relief was provided by subcutaneous administration of 2.5 mg/mL bupivacaine (Hospira Inc., Lake Forest, IL, USA) and 2 mg/kg meloxicam (Felixvet Inc, Kansas City, MO, USA). The dorsal midsection was shaved and disinfected sequentially with Betadine scrubs, povidone-iodine pads, and isopropyl alcohol pads for a total contact time of two minutes. A single 6 mm diameter full-thickness dermal wound was created within this sterile field using a biopsy punch, taking care not to disrupt the underlying musculature. Each wound was inoculated with 10 µL of an *S. aureus* USA300 suspension containing 1 × 10^5^ colony-forming units, and the suspension was pipetted directly onto the wound surface. After infection, animals were placed under a heat source for recovery and maintained either under a heat lamp or on a clean pad over non-electrical heating pads until fully ambulatory. Wounds were topically treated with either sterile water (vehicle), 1× MIC of ceftobiprole, or 1×, 2×, or 5× MIC of NDM-552 every 6 h for a total of 3 days. Mice were euthanized by CO_2_ asphyxiation followed by cervical dislocation. The wound and underlying muscle were excised using an 8 mm diameter biopsy punch and placed into microcentrifuge tubes containing 1 mL of freshly prepared sterile PBS. Tissue samples were homogenized for 3 cycles of 30 s each, serially diluted, and plated to enumerate the bacterial burden.

### 4.13. Checkerboard Assay

Overnight cultures of MRSA ATCC BAA-1556 grown in CAMHB were subcultured to a final concentration of 5 × 10^5^ CFU/mL in CAMHB. All wells of a 96-well plate, except well A1, were inoculated with 100 µL of the bacterial suspension. A 200 µL aliquot of NDM-552 prepared from a 16 mg/mL DMSO stock solution was added to well A1 to achieve a final concentration of 32 µg/mL NDM-552 (0.2% DMSO). Serial two-fold dilutions of NDM-552 were performed across columns by transferring 100 µL sequentially from column A to column H, with mixing five times between each transfer. Column H was reserved for determining the MIC of NDM-552 alone. For combination testing with oxacillin or moenomycin, 100 µL of antibiotic solution (128 µg/mL oxacillin or 0.5 µg/mL moenomycin) was added to wells A1–H1 and serially diluted across the plate. The final row was not mixed and was used to determine the MIC of the antibiotic alone. Plates were sealed with GLAD Press’n Seal and incubated under stationary conditions at 37 °C for 16–18 h. Following incubation, the OD600 of each well was measured. MIC values for NDM-552 alone, each antibiotic alone, and the drug combinations were determined. Fractional inhibitory concentration (FIC) values were calculated as follows: ΣFIC = FIC_NDM-552_ + FIC_antibiotic_, where FIC_NDM-552_ = MIC of NDM-552 in combination/MIC of NDM-552 alone and FIC_antibiotic_ = MIC of antibiotic in combination/MIC of antibiotic alone. Drug interactions were classified as synergistic when ΣFIC ≤ 0.5, additive/indifferent when ΣFIC > 0.5 and <4, and antagonistic when ΣFIC ≥ 4. The assay was performed in three independent replicates.

## 5. Conclusions

In this study, we identify and characterize NDM-552, a novel benzothiophene-containing compound with potent and selective activity against Gram-positive bacteria. Time–kill analyses demonstrate that NDM-552 is bacteriostatic, and cytotoxicity studies reveal a favorable therapeutic index, supporting continued evaluation as an antibacterial lead. Mechanistic investigations indicate that PBP2 is a proposed target of NDM-552. BOCILLIN-FL competition assays and binding studies using MST revealed preferential binding to PBP2, as well as PBP4. We hypothesize that NDM-552 potentially inhibits PBP2 transglycosylation activity due to its unique functionality and necessity for cell survival. NDM-552 shows significant efficacy in a murine wound infection model, achieving reductions in bacterial burden comparable to those of ceftobiprole. This work expands the chemical space of PBP2 inhibitors and provides a strong foundation for further optimization of NDM-552.

## Figures and Tables

**Table 1:** MICs of NDM-552 against Gram-positive bacteria. Control MIC values are provided in Table S6.

Strain	MIC (µg/mL)
MRSA ATCC BAA-1556	1
MRSA ATCC 43300	1
MRSA ATCC BAA-811	1
MRSA ATCC BAA-1753	1
MRSA ATCC BAA-44	1
MRSA ATCC BAA-700789	1
MRSA AH-1263	1
MRSA CRB	1
ATCC MRSA 33591	2
ATCC MRSA BAA-1685	2
ATCC MRSA 700699	2
USA100	1
USA200	1
USA300	2
USA400	1
USA500	1
USA600	1
USA700	1
USA800	1
USA900	1
USA1000	1
USA1100	1
CFSA 1	1
CFSA 2	1
CFSA 3	1
CFSA 4	1
CFSA 5	1
CFSA 6	1
CFSA 7	1
CFSA 8	1
CFSA 9	1
CFSA 12	1
NRS-70	1
MRSA ATCC BAA-1770	1
COL	1
MSSA ATCC 29213	1
*E. faecium* ATCC 51559	1
*S. epidermidis* ATCC 51625	1

**Table 2:** MICs of NDM-552 against Gram-negative bacteria.

Strain	MIC (µg/mL)
KP ATCC BAA-2146	>16
AB ATCC 19606	>16
PAO1	>16
AB 5075	>16
AB 5075 LOS^−^ *	>16
AB 5075 + 60 µM CCCP	>16
AB 5075 + 60 µM PAβN	>16
AB ATCC 17978	>16
AB 17978 ∆*adeIJK*	>16
AB 17978 + 60 µM CCCP	>16
AB 17978 + 60 µM PAβN	>16

* Vancomycin MIC = 1 µg/mL.

**Table 3:** Oxacillin MICs against MRSA 1556^R^ in the presence of NDM-552.

NDM-552 Concentration (µg/mL)	Oxacillin MIC (µg/mL) [Fold Reduction]
0	64
5	0.125 [512]
4	0.125 [512]
3	0.125 [512]
2	0.125 [512]
1	32 [2]

**Table 4:** IC_50_s and K_D_s from BOCILLIN-FL and MST assays for NDM-552 against the five *S. aureus* PBPs.

Protein	IC_50_ from BOCILLIN-FL Assay (µM)	K_D_ from MST Assay (µM)
PBP1	886.1 ± 378.5	51.9 ± 30.4
PBP2	140.1 ± 73.8	12.3 ± 8.5
PBP2a	1985.4 ± 774.5	37.9 ± 20.4
PBP3	5393.9 ± 409.3	81.2 ± 30.2
PBP4	1223.9 ± 297.7	12.0 ± 9.2

**Table 5:** Cytotoxicity and initial pharmacokinetic data.

HepG2 IC_50_ (µM)	Plasma Stability % Remaining 4 h	Cl_int_ (mL/min/kg)	Half-Life (t_1/2_ min)	TI
141.8 ± 9.2	88.2 ± 7.9	224.0 ± 4.0	5.93 ± 0.1	64.8

## Data Availability

The original contributions presented in this study are included in the article/Supplementary Materials. Further inquiries can be directed to the corresponding authors.

## Supplementary Materials

The following supporting information can be downloaded at: https://www.mdpi.com/article/10.3390/antibiotics15090826/s1. References [46,47,48,49,50,51] are cited in the Supplementary Materials.

## Abbreviations

The following abbreviations are used in this manuscript:

AB	Acinetobacter baumannii
ATCC	American Type Culture Collection
BODIPY	Boron dipyrromethene
BPR	Ceftobiprole
CAMHB	Cation-adjusted Mueller-Hinton Broth
CFSA	Cystic fibrosis Staphylococcus aureus
DCM	Dichloromethane
DMF	Dimethylformamide
FBS	Fetal bovine serum
FDAA	Fluorescent D-amino acid
FICI	Fractional inhibitory concentration index
FL	Fluorescent
FoR	Frequency of resistance
KP	Klebsiella pneumoniae
LOD	Limit of detection
LOS	Lipooligosaccharide
MRSA	Methicillin-resistant Staphylococcus aureus
MSSA	Methicillin-susceptible Staphylococcus aureus
MST	Microscale thermophoresis
OD	Optical density
PA	Pseudomonas aeruginosa
PBP	Penicillin-binding protein
PBS	Phosphate-buffered saline
PMBN	Polymyxin B nonapeptide
RT	Room temperature
SD	Standard deviation
SNP	Single nucleotide polymorphism
TEA	Triethylamine
TI	Therapeutic index

## References

[1] Mulani M.S., Kamble E.E., Kumkar S.N., Tawre M.S., Pardesi K.R. (2019). Emerging Strategies to Combat ESKAPE Pathogens in the Era of Antimicrobial Resistance: A Review. Front. Microbiol..

[2] Centers for Disease Control and Prevention (2019). Antibiotic Resistance Threats in the United States, 2019.

[3] CDC (2013). Antibiotic Resistance Threats in the United States, 2013.

[4] Bashabsheh R.H.F., Al-Fawares O., Natsheh I., Bdeir R., Al-Khreshieh R.O., Bashabsheh H.H.F. (2024). epidemiology, pathophysiology, clinical manifestations and application of nano-therapeutics as a promising approach to combat methicillin resistant. Pathog. Glob. Health.

[5] Prickett B. Why Big Pharma Has Abandoned Antibiotics. https://www.nature.com/articles/d41586-020-02884-3.

[6] Lade H., Kim J.S. (2021). Bacterial Targets of Antibiotics in Methicillin-Resistant. Antibiotics.

[7] Sauvage E., Kerff F., Terrak M., Ayala J.A., Charlier P. (2008). The penicillin-binding proteins: Structure and role in peptidoglycan biosynthesis. FEMS Microbiol. Rev..

[8] Chambers H.F., Deleo F.R. (2009). Waves of resistance: Staphylococcus aureus in the antibiotic era. Nat. Rev. Microbiol..

[9] Sauvage E., Terrak M. (2016). Glycosyltransferases and Transpeptidases/Penicillin-Binding Proteins: Valuable Targets for New Antibacterials. Antibiotics.

[10] Baizman E.R., Branstrom A.A., Longley C.B., Allanson N., Sofia M.J., Gange D., Goldman R.C. (2000). Antibacterial activity of synthetic analogues based on the disaccharide structure of moenomycin, an inhibitor of bacterial transglycosylase. Microbiology.

[11] Young M., Walsh D.J., Masters E., Gondil V.S., Laskey E., Klaczko M., Awad H., McGrath J., Schwarz E.M., Melander C. (2022). Identification of Identification of *Staphylococcus aureus* Penicillin Binding Protein 4 (PBP4) inhibitors. Antibiotics.

[12] Stefaniak M.A., Gondil V.S., Gillis E.P., Nemeth A.M., Oliver A.G., Melander R.J., Dunman P.M., Melander C. (2026). Exploration of a benzothiophene scaffold for use as adjuvants with β-lactam antibiotics against methicillin-resistant. RSC Med. Chem..

[13] Moffatt J.H., Harper M., Harrison P., Hale J.D., Vinogradov E., Seemann T., Henry R., Crane B., St Michael F., Cox A.D. (2010). Colistin resistance in Acinetobacter baumannii is mediated by complete loss of lipopolysaccharide production. Antimicrob. Agents Chemother..

[14] Powers M.J., Trent M.S. (2018). Phospholipid retention in the absence of asymmetry strengthens the outer membrane permeability barrier to last-resort antibiotics. Proc. Natl. Acad. Sci. USA.

[15] Amaral L., Martins A., Spengler G., Molnar J. (2014). Efflux pumps of Gram-negative bacteria: What they do, how they do it, with what and how to deal with them. Front. Pharmacol..

[16] Darby E.M., Bavro V.N., Dunn S., McNally A., Blair J.M.A. (2023). RND pumps across the genus. Microb. Genom..

[17] Osei Sekyere J., Amoako D.G. (2017). Carbonyl Cyanide m-Chlorophenylhydrazine (CCCP) Reverses Resistance to Colistin, but Not to Carbapenems and Tigecycline in Multidrug-Resistant. Front. Microbiol..

[18] Lamers R.P., Cavallari J.F., Burrows L.L. (2013). The efflux inhibitor phenylalanine-arginine beta-naphthylamide (PAβN) permeabilizes the outer membrane of gram-negative bacteria. PLoS ONE.

[19] Tsubery H., Ofek I., Cohen S., Fridkin M. (2000). Structure-function studies of polymyxin B nonapeptide: Implications to sensitization of gram-negative bacteria. J. Med. Chem..

[20] Melander R.J., Mattingly A.E., Nemeth A.M., Melander C. (2022). Overcoming intrinsic resistance in gram-negative bacteria using small molecule adjuvants. Bioorg Med. Chem. Lett..

[21] Vaara M. (2019). Polymyxin Derivatives that Sensitize Gram-Negative Bacteria to Other Antibiotics. Molecules.

[22] Ishak A., Mazonakis N., Spernovasilis N., Akinosoglou K., Tsioutis C. (2025). Bactericidal versus bacteriostatic antibacterials: Clinical significance, differences and synergistic potential in clinical practice. J. Antimicrob. Chemother..

[23] Li J., Xie S., Ahmed S., Wang F., Gu Y., Zhang C., Chai X., Wu Y., Cai J., Cheng G. (2017). Antimicrobial Activity and Resistance: Influencing Factors. Front. Pharmacol..

[24] Auld D.S., Inglese J., Dahlin J.L., Sittampalam G.S., Coussens N.P., Brimacombe K., Grossman A., Arkin M., Auld D., Austin C., Baell J., Bejcek B., Caaveiro J.M.M. (2004). Assay Interference by Aggregation. Assay Guidance Manual.

[25] He X., Xv S., Liu R., Duan M., Fan W., Fan B. (2024). Triton X-100 counteracts antibiotic resistance of Enterococcus faecalis: An in vitro study. J. Dent..

[26] Chraibi M., Farah A., Elamin O., Iraqui H.M., Fikri-Benbrahim K. (2020). Characterization, antioxidant, antimycobacterial, antimicrobial effcts of Moroccan rosemary essential oil, and its synergistic antimicrobial potential with carvacrol. J. Adv. Pharm. Technol. Res..

[27] Rosche W.A., Foster P.L. (2000). Determining mutation rates in bacterial populations. Methods.

[28] Lang G.I. (2018). Measuring Mutation Rates Using the Luria-Delbrück Fluctuation Assay. Genome Instability: Methods and Protocols.

[29] Giulieri S.G., Guérillot R., Kwong J.C., Monk I.R., Hayes A.S., Daniel D., Baines S., Sherry N.L., Holmes N.E., Ward P. (2020). Comprehensive Genomic Investigation of Adaptive Mutations Driving the Low-Level Oxacillin Resistance Phenotype in Staphylococcus aureus. mBio.

[30] Shirley J.D., Gillingham J.R., Nauta K.M., Diwakar S., Carlson E.E. (2024). k_inact_/K_I_ Value Determination for Penicillin-Binding Proteins in Live Cells. ACS Infect. Dis..

[31] Bouley R., Kumarasiri M., Peng Z., Otero L.H., Song W., Suckow M.A., Schroeder V.A., Wolter W.R., Lastochkin E., Antunes N.T. (2015). Discovery of antibiotic (E)-3-(3-carboxyphenyl)-2-(4-cyanostyryl)quinazolin-4(3H)-one. J. Am. Chem. Soc..

[32] Otero L.H., Rojas-Altuve A., Llarrull L.I., Carrasco-López C., Kumarasiri M., Lastochkin E., Fishovitz J., Dawley M., Hesek D., Lee M. (2013). How allosteric control of Staphylococcus aureus penicillin binding protein 2a enables methicillin resistance and physiological function. Proc. Natl. Acad. Sci. USA.

[33] Cheng T.J., Sung M.T., Liao H.Y., Chang Y.F., Chen C.W., Huang C.Y., Chou L.Y., Wu Y.D., Chen Y.H., Cheng Y.S. (2008). Domain requirement of moenomycin binding to bifunctional transglycosylases and development of high-throughput discovery of antibiotics. Proc. Natl. Acad. Sci. USA.

[34] Peters K., Pazos M., VanNieuwenhze M.S., Vollmer W. (2019). Optimized Protocol for the Incorporation of FDAA (HADA Labeling) for in situ Labeling of Peptidoglycan. Bio Protoc..

[35] Pinho M.G., Errington J. (2005). Recruitment of penicillin-binding protein PBP2 to the division site of Staphylococcus aureus is dependent on its transpeptidation substrates. Mol. Microbiol..

[36] Punekar A.S., Samsudin F., Lloyd A.J., Dowson C.G., Scott D.J., Khalid S., Roper D.I. (2018). The role of the jaw subdomain of peptidoglycan glycosyltransferases for lipid II polymerization. Cell Surf..

[37] Sardis M.F., Bohrhunter J.L., Greene N.G., Bernhardt T.G. (2021). The LpoA activator is required to stimulate the peptidoglycan polymerase activity of its cognate cell wall synthase PBP1a. Proc. Natl. Acad. Sci. USA.

[38] Egan A.J.F., Maya-Martinez R., Ayala I., Bougault C.M., Banzhaf M., Breukink E., Vollmer W., Simorre J.P. (2018). Induced conformational changes activate the peptidoglycan synthase PBP1B. Mol. Microbiol..

[39] Kim C., Milheiriço C., Gardete S., Holmes M.A., Holden M.T., de Lencastre H., Tomasz A. (2012). Properties of a novel PBP2A protein homolog from Staphylococcus aureus strain LGA251 and its contribution to the β-lactam-resistant phenotype. J. Biol. Chem..

[40] Pinho M.G., de Lencastre H., Tomasz A. (1998). Transcriptional analysis of the Staphylococcus aureus penicillin binding protein 2 gene. J. Bacteriol..

[41] Guignard B., Majcherczyk P.A., Monachon C., Vouillamoz J., Moreillon P. (2017). Muropeptide signature of inhibitors of protein synthesis correlates with β-lactam synergism against methicillin-resistant Staphylococcus aureus. Int. J. Antimicrob. Agents.

[42] Adedeji-Olulana A.F., Wacnik K., Lafage L., Pasquina-Lemonche L., Tinajero-Trejo M., Sutton J.A.F., Bilyk B., Irving S.E., Portman Ross C.J., Meacock O.J. (2024). Two codependent routes lead to high-level MRSA. Science.

[43] da Costa T.M., de Oliveira C.R., Chambers H.F., Chatterjee S.S. (2018). PBP4: A New Perspective on Staphylococcus aureus β-Lactam Resistance. Microorganisms.

[44] O’Daniel P.I., Peng Z., Pi H., Testero S.A., Ding D., Spink E., Leemans E., Boudreau M.A., Yamaguchi T., Schroeder V.A. (2014). Discovery of a new class of non-β-lactam inhibitors of penicillin-binding proteins with Gram-positive antibacterial activity. J. Am. Chem. Soc..

[45] Gondil V.S., Butman H.S., Young M., Walsh D.J., Narkhede Y., Zeiler M.J., Crow A.H., Carpenter M.E., Mardikar A., Melander R.J. (2024). Development of phenyl-urea-based small molecules that target penicillin-binding protein 4. Chem. Biol. Drug Des..

[46] Tenover Fred C., McDougal Linda K., Goering Richard V., Killgore G., Projan Steven J., Patel Jean B., Dunman Paul M. (2006). Characterization of a Strain of Community-Associated Methicillin-Resistant *Staphylococcus aureus* Widely Disseminated in the UnitedStates. J. Clin. Microbiol..

[47] Yang H., Wang X., Wang C., Yin F., Qu L., Shi C., Zhao J., Li S., Ji L., Peng W. (2021). Optimization of WZ4003 as NUAK inhibitors against human colorectal cancer. Eur. J. Med. Chem..

[48] Bruker AXS (2023). APEX-5. https://www.bruker.com/it/products-and-solutions/diffractometers-and-x-ray-microscopes/single-crystal-x-ray-diffractometers/sc-xrd-software.html.

[49] Krause L., Herbst-Irmer R., Sheldrick G.M., Stalke D. (2015). Comparison of silver and molybdenum microfocus X-ray sources for single-crystal structure determination. J. Appl. Crystallogr..

[50] Sheldrick G.M. (2015). Crystal structure refinement with SHELXL. Acta Crystallogr. C Struct. Chem..

[51] Sheldrick G.M. (2015). SHELXT—Integrated space-group and crystal-structure determination. Acta Crystallogr. A Found. Adv..

