# Supplementary material for: Discovery of a Novel Benzothiophene Inhibitor of Penicillin-Binding Protein 2 with Potent Gram-Positive Activity
Source: Antibiotics (Basel). 2026 Aug 25;15(9):826. doi: 10.3390/antibiotics15090826 (PMC13603487; doi:10.3390/antibiotics15090826)
Supplement: Supplementary file 1 [file antibiotics-15-00826-s001.zip › antibiotics-4468723-supplementary.pdf]

SUPPORTING INFORMATION

Discovery and evaluation of a novel class of benzothiophene-containing inhibitors of Penicillin-Binding Protein 2 with Broad spectrum gram-positive antibacterial activity

Monica A. Stefaniak[1], Vijay Singh Gondil[2], Jingdong Yang[1], Adil Omar[1], Allen G. Oliver[1], Ansley M. Nemeth[1], Roberta J. Melander[1], Mayland Chang[1], Paul M. Dunman,[2*] Christian Melander[1*]

[1]Department of Chemistry and Biochemistry, University of Notre Dame, Notre Dame, IN, 46556, United States.
[2]Department of Microbiology and Immunology, University of Rochester Medical Center, Rochester, New York 14642, United States.

*Corresponding authors E-mail address: paul_dunman@urmc.rochester.edu and cmelande@nd.edu

Table of Contents

Bacterial strains, media, and additional culture conditions 2

Biological Assay Protocols 3

Supplemental Figures 3

Table S1. MIC of NDM-552 in MRSA ATCC 1556 with and without addition of FBS and Triton-X 3
Table S2. Checkerboard assay in MRSA 1556 between NDM-552 and oxacillin. 3
Table S3. MIC of NDM-552 every second day of evolution experiment. 3
Table S4. Potentiation of azithromycin (AZM), bacitracin (BAC), and tetracycline (TET) with 2 µg/mL of NDM-552 in the evolved ATCC MRSA 1556 strain. 3
Table S5. Checkerboard assay in MRSA 1556 between NDM-552 and moenomycin. 3
Table S6. Antibiotic control MIC values for strains tested. 4

General Chemistry Experimental 5

Previously Reported Compounds 6

Novel Compounds 7

^{1}H NMR and ^{13}C NMR spectra of novel compound 8

References 10

X-ray Crystallographic Data for NDM-552 (Tables S7-S13) 11

General Biological Experimental

Bacterial strains, media, and additional culture conditions

MRSA ATCC BAA-1556, MRSA ATCC 43300, MRSA ATCC BAA-811, MRSA ATCC BAA-1753, MRSA ATCC BA-44, MRSA ATCC BAA-700789, MRSA ATCC 33591, MRSA ATCC 1685, MRSA ATCC 700699, MSSA ATCC 29213, MRSA ATCC BAA-1770, *E. faecium* ATCC 51559, *S. epidermidis* ATCC 51625, AB 19606 and KP 2146 were obtained from the American Type Culture Collection (ATCC). MRSA AH-1263 and MRSA NRS-70 were obtained from Professor Alexander Horswill at the University of Colorado Anschutz Medical Campus. Cystic fibrosis *S. aureus* strains (CFSA) 1 – 9 and CFSA 12 were obtained from Professor Pradeep Singh at the University of Washington. USA100 – USA1100 were obtained from the CDC strain collection.[1] *A. baumannii* 17978 wild-type and *ΔadeIJK* strains were obtained from Professor Edward Geisinger at Northeastern University. PA O1 was obtained from Professor Colin Manoil at the University of Washington. CRB bacterial strain was kindly gifted by Dr. Som S. Chatterjee, University of Maryland, USA. Stock cultures were stored in 25% glycerol and maintained at -80 °C. Prior to use, colonies were grown on LB (Lennox) (Research Products International, catalog # L24060-2000.0) agar (Fisher, catalog #BP1423-500) (*P. aeruginosa, A. baumannii,* and *K. pneumoniae)*, tryptic soy agar (Sigma Aldrich, catalog # BCCM0191) (*S. aureus*), or Mueller-Hinton broth (MHB) (Becton Dickinson, catalog #0344C30) (CRB, COL). Cation-adjusted Mueller Hinton broth (CAMHB) (Becton Dickinson, catalog # 0190M57). Oxacillin sodium salt monohydrate (catalog #O0353) was purchased from TCI Chemicals. Azithromycin dihydrate (catalog #A2076) was obtained from TCI. Bacitracin (catalog #11702) was obtained from Sigma Aldrich. Tetracycline hydrochloride (catalog #T7660) was obtained from Sigma Aldrich. Ceftobiprole was purchased from Medchemexpress (catalog #HY-112579) and dissolved in DMSO. **NDM-552** was dissolved in molecular biology grade DMSO (MP Biomedicals, catalog #194819) as an 8 mg/mL stock and stored at -20 °C until further use. Oxacillin, bacitracin, and tetracycline were dissolved in sterile Milli-Q water. Azithromycin was dissolved in DMSO. Fetal bovine serum (FBS) was purchased from VWR (catalog # 89510-182). Triton-X detergent was purchased from Acros Organics (catalog #327371000). Tween-20 was purchased from Sigma Aldrich (catalog #P5927).

Biological Assay Protocols

Supplemental Figures

Table S1. MIC of NDM-552 in MRSA ATCC 1556 with and without addition of FBS and Triton-X

Condition	MIC **NDM-552** (µg/mL)
CAMHB	1
9:1 CAMHB:FBS	16
0.01% Triton-X	1

Table S2. Checkerboard assay in MRSA 1556 between NDM-552 and oxacillin.

**MIC NDM-552 [MIC Combo]**	**MIC OX [MIC combo]**	**FICI**
1 [0.5]	32 [8]	0.75 (Additive)

Concentrations in µg/mL. $FIC_{compound}$ = MIC of compound in combination/MIC of compound alone. $FIC_{oxacillin}$ = MIC of oxacillin in combination/MIC of oxacillin alone. FICI was calculated as FICI = $FIC_{compound}$ + $FIC_{oxacillin}$. Synergistic when FICI = ≤ 0.5, additive when FICI is between 0.5 and 1, antagonistic when FICI = ≥ 2.

Table S3. MIC of NDM-552 every second day of evolution experiment.

-	MIC **NDM-552** (µg/mL)
Day 0	1
Day 2	64
Day 4	>64
Day 6	>64
Day 8	>64

Table S4. Potentiation of azithromycin (AZM), bacitracin (BAC), and tetracycline (TET) with 2 µg/mL of NDM-552 in the evolved ATCC MRSA 1556 strain.

**MRSA 1556^{R}**		
**Standalone MIC NDM-552 (µg/mL)**	**Concentration (µg/mL)**	**AZM MIC (µg/mL) [fold reduction]**
	**-**	64
>64	2	64 [-]
		**BAC MIC (µg/mL) [fold reduction]**
	-	16
	2	16 [-]
		**TET MIC (µg/mL) [fold reduction]**
	-	32
	2	8 [4]
		**IMI MIC (µg/mL) [fold reduction]**

Table S5. Checkerboard assay in MRSA 1556 between NDM-552 and moenomycin.

**MIC NDM-552 [MIC Combo]**	**MIC Moenomycin [MIC combo]**	**FICI**
1 [0.25]	0.25 [0.125]	0.75 (Additive)

Concentrations in µg/mL. $FIC_{compound}$ = MIC of compound in combination/MIC of compound alone. $FIC_{moenomycin}$ = MIC of colistin in combination/MIC of moenomycin alone. FICI was calculated as FICI = $FIC_{compound}$ +

$FIC_{moenomycin}$. Synergistic when FICI = ≤ 0.5, additive when FICI is between 0.5 and 1, antagonistic when FICI = ≥ 2.

Table S6. Antibiotic control MIC values for strains tested.

Strain	Antibiotic MIC (µg/mL)
-	Oxacillin
MRSA ATCC BAA-1556	32
MRSA ATCC 43300	32
MRSA ATCC BAA-811	64
MRSA ATCC BAA-1753	256
MRSA ATCC BAA-44	512
MRSA ATCC BAA-700789	256
MRSA AH-1263	32
ATCC MRSA 33591	128
ATCC MRSA BAA-1685	256
ATCC MRSA 700699	512
USA100	16
USA200	>256
USA300	8
USA400	2
USA500	32
USA600	64
USA700	8
USA800	2
USA900	0.5
USA1000	32
USA1100	16
CFSA 1	1
CFSA 2	64
CFSA 3	0.25
CFSA 4	0.5
CFSA 5	1024
CFSA 6	0.25
CFSA 7	128
CFSA 8	0.5
CFSA 9	0.5
CFSA 12	0.25
NRS-70	>512
MRSA ATCC BAA-1770	32
COL	256
MSSA ATCC 29213	0.25
S. epidermidis ATCC 51625	>256
-	Vancomycin MIC (µg/mL)
E. faecium ATCC 51559	>256
	Ceftobiprole MIC (µg/mL)
MRSA CRB	128
-	Colistin MIC (µg/mL)
KP ATCC BAA-2146	2
AB ATCC 19606	2
PAO1	2
AB 5075	2
AB 5075 LOS^{-}	128
AB ATCC 17978	2

AB 17978 Δ*adeIJK*	2

General Chemistry Experimental

All reactions were carried out under an atmosphere of nitrogen, unless otherwise noted. All chemical reagents and anhydrous solvents for synthesis were purchased from commercial sources (VWR, Sigma-Aldrich, Fischer Scientific, Ambeed, TCI, or Acros Organics) and used without further purification. Reactions were monitored via thin layer chromatography (TLC) using glass-backed pre-coated silica gel plates from VWR (TLC Silica Gel 60 Sheets, MilliporeSigma, F254, 60 Å pore, 230–400 mesh) and visualized with 254 nM UV light. Column chromatography was performed using 60 Å mesh, particle size 40–60 µm, standard grade silica gel from Sorbent Technologies. NMR solvents were purchased from MilliporeSigma, BeanTown Chemical, or Thermo Scientific, and used without further purification. All ^{1}H NMR (400 MHz or 500 MHz) were recorded at 25 °C on Bruker AVANCE III HD spectrometers. All ^{13}C NMR (101 MHz or 126 MHz) were recorded at 25 °C on Bruker AVANCE III HD spectrometers. Chemical shifts (δ) are reported in parts per million (ppm) relative to the respective NMR solvent; coupling constants (*J*) are in hertz (Hz). Abbreviations used are s, singlet; d, doublet; dd, doublet of doublets; dt, doublet of triplets; t, triplet; q, quartet; m, multiplet; br, broad. High-resolution mass spectrometry (HRMS) measurements were determined by electrospray ionization (ESI) on a LC/ESI-QTOF-MS system (Thermo Scientific UltiMate 3000 UHPLC system coupled to a Bruker micrOTOF-Q II quadrupole time-of-flight mass spectrometer, ESI-QqTOF-MS) at the Mass Spectrometry and Proteomics Facility at the University of Notre Dame. Infrared spectra were recorded on a Bruker Alpha II FT-IR spectrometer (ν_{max} in cm^{-1}). UV-Vis absorbance measurements were recorded on a Thermo Spectronic Genesys 10 UV-Vis scanning spectrophotometer and reported as the wavelength of maximum absorbance (λ_{max} in nm). Purities of tested compounds were all verified to be ≥95% by LC-MS analysis on a Advion AVANT Expression Model L LC/CMS (compact mass spectrometer) system (Kinetex Polar C18 column, 2.6 µm particle size, 50 x 2.1 mm) or a Thermo Scientific UltiMate 3000 UHPLC system.

Previously Reported Compounds

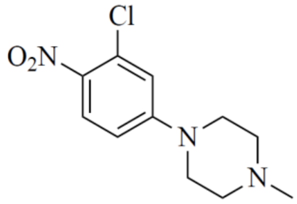

1-(3-chloro-4-nitrophenyl)-4-methylpiperazine (2)

1-(3-chloro-4-nitrophenyl)-4-methylpiperazine was prepared from 2-chloro-4-fluoro-1-nitrobenzene and 1-methylpiperazine.[2] To a flame dried round-bottom flask, 2-chloro-4-fluoro-1-nitrobenzene (1 g, 6 mmol), 1-methylpiperazine (700 mg, 7 mmol), and potassium carbonate (2 g, 10 mmol) were dissolved in anhydrous DMF (5 mL). The reaction mixture was stirred at room temperature for 30 minutes, or when starting material was consumed via TLC. The resulting mixture was diluted with chloroform, washed with brine (3 X 10 mL), dried using sodium sulfate, filtered, and concentrated under reduced pressure. Compound **2** was obtained as an orange solid in 95.3% yield (953 mg, 3.73 mmol). ^{1}H NMR (400 MHz, $CDCl_3$) δ 8.02 (d, J = 9.3 Hz, 1H), 6.85 (d, J = 2.8 Hz, 1H), 6.73 (dd, J = 9.4, 2.8 Hz, 1H), 3.45 – 3.38 (m, 4H), 2.58 – 2.51 (m, 4H), 2.36 (s, 3H). Spectral data were consistent with previously reported values.[2]

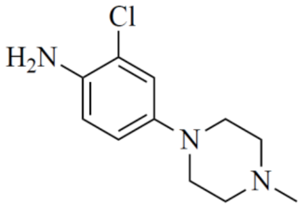

2-chloro-4-(4-methylpiperazin-1-yl)aniline (3)

2-chloro-4-(4-methylpiperazin-1-yl)aniline was prepared from 1-(3-chloro-4-nitrophenyl)-4-methylpiperazine as previously described.[2] To a flame dried round-bottom flask, 1-(3-chloro-4-nitrophenyl)-4-methylpiperazine (500 mg, 1.96 mmol), tin(II) chloride (2.22 g, 11.7 mmol), and concentrated HCl (713 mg, 19.6 mmol) were dissolved in ethanol (10 mL). The reaction mixture was heated to 80 °C for one hour, or when starting material was consumed via TLC. Once cooled, the reaction mixture was concentrated under reduced pressure and neutralized to pH 7 with sodium bicarbonate. The mixture washed with saturated aqueous sodium bicarbonate (3 x 15 mL) and brine (1 x 15 mL). The organic layer was dried with sodium sulfate and concentrated under vacuum to provide 2-chloro-4-(4-methylpiperazin-1-yl)aniline as a white solid in 92.7% yield (409 mg, 1.81 mmol). ^{1}H NMR (400 MHz, DMSO-d_6) δ 6.76 (d, J = 2.4 Hz, 1H), 6.73–6.71 (m, 2H), 4.78 (s, 2H), 2.92 (t, J = 4.9 Hz, 4H), 2.41 (t, J = 4.9 Hz, 4H), 2.19 (s, 3H). Spectral data were consistent with previously reported values.[2]

Novel Compounds

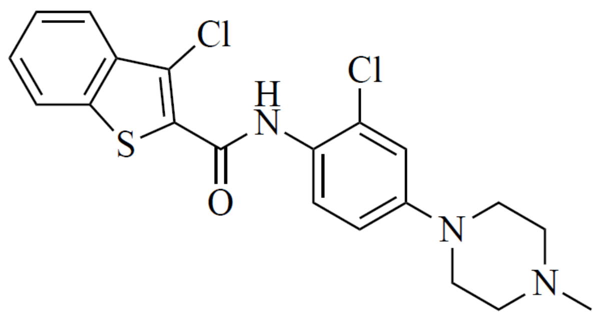

3-chloro-*N*-(2-chloro-4-(4-methylpiperazin-1-yl)phenyl)benzo[*b*]thiophene-2-carboxamide (**NDM-552)**

3-chloro-*N*-(2-chloro-4-(4-methylpiperazin-1-yl)phenyl)benzo[*b*]thiophene-2-carboxamide was prepared from 2-chloro-4-(4-methylpiperazin-1-yl)aniline and 3-chlorobenzothiophene-2-carbonyl chloride. To a flame-dried round-bottom flask, 3-chlorobenzothiophene-2-carbonyl chloride (187 mg, 877 µmol), 2-chloro-4-(4-methylpiperazin-1-yl)aniline (180 mg, 797 µmol), and TEA (121 mg, 1.20 mmol) were dissolved in anhydrous DCM (5 mL). The reaction mixture was stirred at room temperature for 16 hr. The mixture was washed once with sodium bicarbonate, dried with sodium sulfate, filtered, and solvent was evaporated under reduced pressure. The crude material was purified by flash column chromatography using 10% methanol in chloroform to afford **NDM-552** in 33.7 % yield (113 mg, 269 µmol). The final compound was washed with methanol and concentrated HCl to obtain the compound as an HCl salt. ^{1}H NMR (400 MHz, CDCl3) δ 9.38 (s, 1H), 8.44 (d, J = 9.2 Hz, 1H), 7.99 – 7.86 (m, 2H), 7.59 – 7.52 (m, 2H), 7.01 (d, J = 2.8 Hz, 1H), 6.92 (dd, J = 9.0, 2.8 Hz, 1H), 3.27 (s, 4H), 2.64 (s, 4H), 2.41 (s, 3H). ^{13}C NMR (101 MHz, DMSO-d_6) δ 159.4, 150.4, 137.4, 136.5, 132.6, 129.0, 128.2, 127.5, 126.6, 125.2, 124.0, 123.2, 119.9, 115.6, 114.5, 54.8, 49.1, 48.1, 46.2. HRMS (ESI) *m/z* calculated for $C_{20}H_{20}Cl_2N_3OS$ [M + H]$^+$: 420.0699, found 420.0682. LC trace: 97.78%, UV (λ_{max}): 286 nm. IR ν_{max} (cm^{-1}): 3350, 2362, 1657.

^{1}H NMR and ^{13}C NMR spectra of novel compound

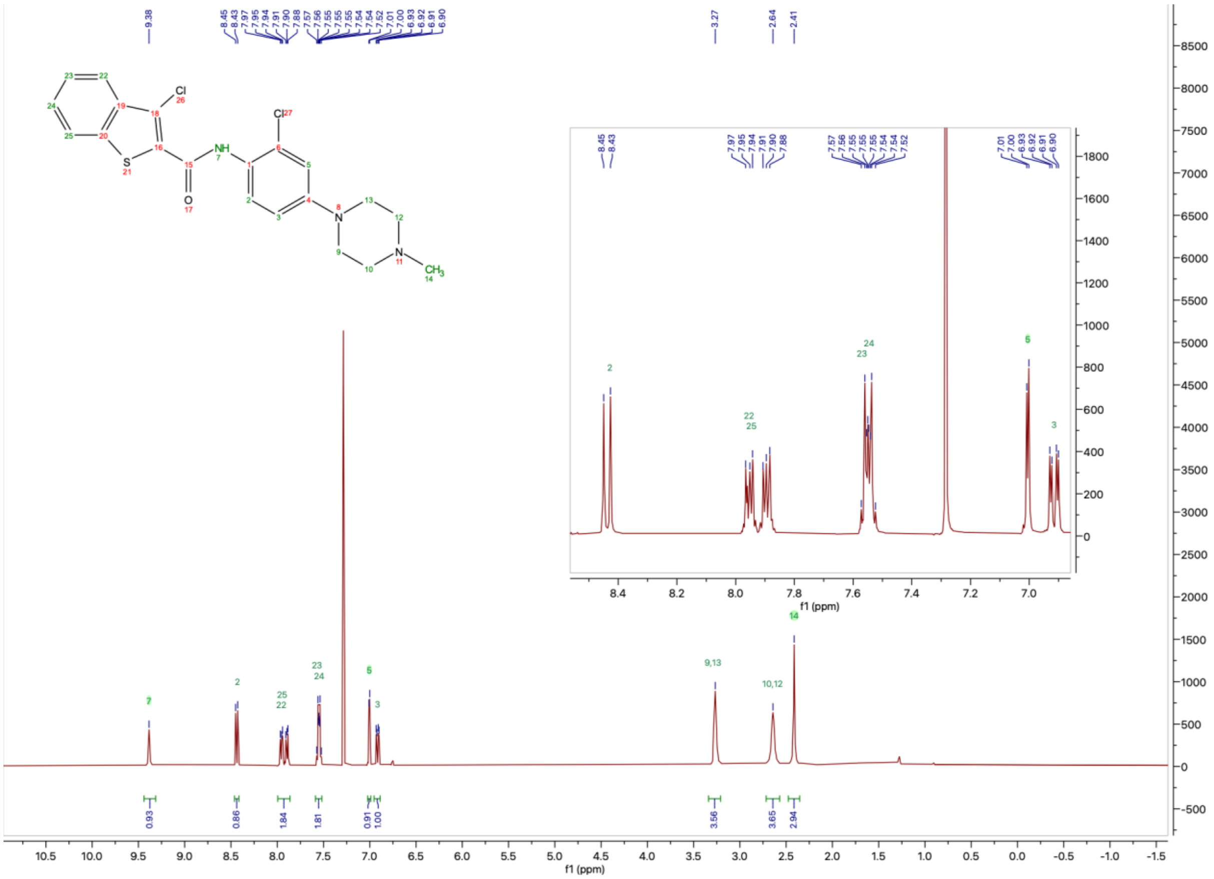

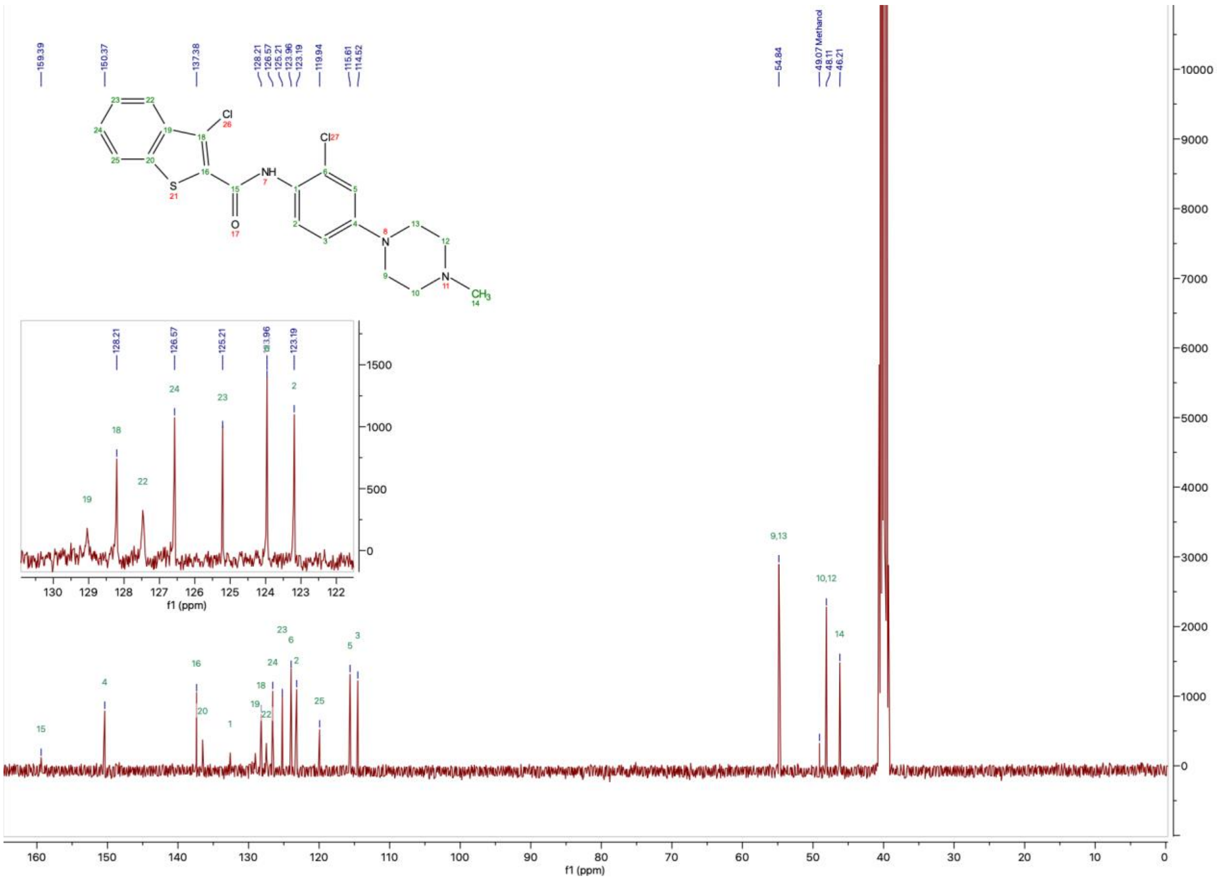

159.39
150.37
137.38
128.21
126.57
125.21
123.96
123.19
119.94
115.61
114.52
54.84
49.07 Methanol
48.11
46.21
Cl
NH
S
O
CH3
f1 (ppm)

References

(1) Tenover Fred, C.; McDougal Linda, K.; Goering Richard, V.; Killgore, G.; Projan Steven, J.; Patel Jean, B.; Dunman Paul, M. Characterization of a Strain of Community-Associated Methicillin-Resistant *Staphylococcus aureus* Widely Disseminated in the UnitedStates. *J Clin Microbiol* **2006**, *44*. DOI: https://doi.org/10.1128/jcm.44.1.108-118.2006.
(2) Yang, H.; Wang, X.; Wang, C.; Yin, F.; Qu, L.; Shi, C.; Zhao, J.; Li, S.; Ji, L.; Peng, W.; et al. Optimization of WZ4003 as NUAK inhibitors against human colorectal cancer. *Eur J Med Chem* **2021**, *210*, 113080. DOI: 10.1016/j.ejmech.2020.113080.
(3) *Bruker AXS*. *APEX-5*, 2023. https://www.bruker.com/en/products-and-solutions/diffractometers-and-x-ray-microscopes/single-crystal-x-ray-diffractometers/sc-xrd-software/apex.html.
(4) Krause, L.; Herbst-Irmer, R.; Sheldrick, G. M.; Stalke, D. Comparison of silver and molybdenum microfocus X-ray sources for single-crystal structure determination. *J Appl Crystallogr* **2015**, *48* (Pt 1), 3-10. DOI: 10.1107/S1600576714022985.
(5) Sheldrick, G. M. Crystal structure refinement with SHELXL. *Acta Crystallogr C Struct Chem* **2015**, *71* (Pt 1), 3-8. DOI: 10.1107/S2053229614024218.
(6) Sheldrick, G. M. SHELXT - integrated space-group and crystal-structure determination. *Acta Crystallogr A Found Adv* **2015**, *71* (Pt 1), 3-8. DOI: 10.1107/S2053273314026370.

X-ray Crystallographic Data for NDM-552 (Tables S7-S13)

DISCUSSION

The organic chloride salt crystallizes as yellow needle-like crystals from a methanol/water solution. There is one molecule of the organic cation compound, one chloride and one modeled methanol of crystallization in the asymmetric unit of the primitive, centrosymmetric, monoclinic space group P-1. The model was treated with the SQUEEZE routine implemented in PLATON [5]. This accounted for 29 electrons in a 111 $Å^3$ void in the crystal lattice. This corresponds closely to an additional methanol (14 e^-) and water of crystallization (10 e^-) per unit cell. As such this has been added as an additional half molecule of methanol and water per asymmetric unit to the chemical formula.

The structure of the organic cation is as expected (see Figures). Amide nitrogen, N1, is located that it forms bifurcated hydrogen bonds to the chlorine atoms within the cation (see Table of Hydrogen Bonds). Amido nitrogen, N3, forms a hydrogen bond to the nearby chloride counterion. The located methanol also forms a hydrogen bond to that chlorine atom.

Bond distances and angles within the molecules are otherwise as expected.

ACKNOWLEDGMENT

Funding for instrumentation used in this study was supported by NSF MRI Award CHE-2214606.

CRYSTAL SUMMARY

Crystal data for $C_{21.50}H_{27}Cl_3N_3O_3S$; M_r = 513.87; Triclinic; space group P-1; a = 9.2168(8) Å; b = 9.5677(8) Å; c = 15.0458(13) Å; α = 88.342(3)°; β = 73.598(3)°; γ = 67.196(3)°; V = 1168.44(18) $Å^3$; Z = 2; T = 120(2) K; λ(Cu-Kα) = 1.54178 Å; μ(Cu-Kα) = 4.634 mm^{-1}; d_{calc} = 1.461 $g.cm^{-3}$; 43957 reflections collected; 4771 unique (R_{int} = 0.0330); giving R_1 = 0.0538, wR_2 = 0.1349 for 4659 data with [I>2σ(I)] and R_1 = 0.0547, wR_2 = 0.1355 for all 4771 data. Residual electron density ($e^-.Å^{-3}$) max/min: 2.108/-1.282.

An arbitrary sphere of data was collected on a yellow, needle-like crystal, having approximate dimensions of 0.291 × 0.076 × 0.058 mm, on a Bruker Venture diffractometer equipped with a Bruker PHOTON-III detector using a combination of ω- and φ-scans of 0.5°.[3] Data were corrected for absorption and polarization effects and analyzed for space group determination.[4] The structure was solved by dual-space methods and expanded routinely.[5] The model was refined by full-matrix least-squares analysis of F^2 against all reflections.[6] All non-hydrogen atoms were refined with anisotropic atomic displacement parameters. Unless otherwise noted, hydrogen atoms were included in calculated positions. Atomic displacement parameters for the hydrogens were tied to the equivalent isotropic displacement parameter of the atom to which they are bonded (U_{iso}(H) = 1.5U_{eq}(C) for methyl, 1.2U_{eq}(C) for all others).

Table S7. Crystal data and structure refinement for ndm552_sq.

Identification code	ndm552_sq
Empirical formula	$C_{21.50}H_{27}Cl_3N_3O_3S$
Formula weight	513.87
Temperature (K)	120(2)
Wavelength (Å)	1.54178
Crystal system	Triclinic
Space group	P-1
Unit cell dimensions	
a (Å)	9.2168(8)
b (Å)	9.5677(8)
c (Å)	15.0458(13)
α (°)	88.342(3)
β (°)	73.598(3)
γ (°)	67.196(3)
Volume (Å^3)	1168.44(18)
Z	2
Density (calculated, g.cm^{-3})	1.461
Absorption coefficient (μ, mm^{-1})	4.634
F(000)	536
Crystal color, habit	yellow, needle
Crystal size (mm^3)	0.291 × 0.076 × 0.058
θ range for data collection (°)	3.074 to 74.976
Index ranges	$-11 \leq h \leq 11$, $-11 \leq k \leq 11$, $-18 \leq l \leq 18$
Reflections collected	43957
Independent reflections	4771 [R_{int} = 0.0330]
Completeness to θ = 67.679°	100.0 %
Absorption correction	Numerical
Max. and min. transmission	0.7419 and 0.3752
Refinement method	Full-matrix least-squares on F^2
Data / restraints / parameters	4771 / 0 / 282
Goodness-of-fit on F^2	1.047
Final R indices [I>2σ(I)]	R_1 = 0.0538, wR_2 = 0.1349
R indices (all data)	R_1 = 0.0547, wR_2 = 0.1355
Extinction coefficient	n/a
Largest diff. peak and hole (e$^-$.Å^{-3})	2.108 and -1.282

Table S8. Atomic coordinates and equivalent isotropic displacement parameters (Å^2) for ndm552_sq. U(eq) is defined as one third of the trace of the orthogonalized U_{ij} tensor.

	x	y	z	U(eq)
Cl(1)	0.20768(8)	0.95755(7)	0.48037(4)	0.022(1)
Cl(2)	0.45400(8)	0.60759(8)	0.33012(5)	0.027(1)
S(1)	0.41295(8)	1.01498(7)	0.69066(4)	0.020(1)
O(1)	0.6598(2)	0.7361(2)	0.59335(14)	0.026(1)
N(1)	0.5432(3)	0.7011(3)	0.48442(16)	0.019(1)
N(2)	1.0093(3)	0.1508(3)	0.27227(16)	0.019(1)
N(3)	1.2562(3)	-0.0751(3)	0.12598(16)	0.020(1)
C(1)	0.2263(3)	1.1648(3)	0.69852(19)	0.019(1)
C(2)	0.1462(4)	1.2918(3)	0.76410(19)	0.022(1)
C(3)	-0.0020(4)	1.3981(3)	0.7587(2)	0.023(1)
C(4)	-0.0720(3)	1.3813(3)	0.6902(2)	0.024(1)
C(5)	0.0061(3)	1.2554(3)	0.62570(19)	0.021(1)
C(6)	0.1587(3)	1.1451(3)	0.62947(18)	0.019(1)
C(7)	0.2638(3)	1.0066(3)	0.57123(18)	0.019(1)
C(8)	0.4035(3)	0.9247(3)	0.59460(18)	0.019(1)
C(9)	0.5477(3)	0.7784(3)	0.55754(19)	0.019(1)
C(10)	0.6622(3)	0.5626(3)	0.43464(18)	0.018(1)
C(11)	0.6342(3)	0.5054(3)	0.35978(19)	0.019(1)
C(13)	0.8919(3)	0.2887(3)	0.32492(19)	0.019(1)
C(14)	0.9213(3)	0.3441(3)	0.40055(19)	0.021(1)
C(15)	0.8099(3)	0.4774(3)	0.45419(19)	0.020(1)
C(12)	0.7443(3)	0.3723(3)	0.30541(19)	0.020(1)
C(16)	0.9648(3)	0.0996(3)	0.1969(2)	0.022(1)
C(17)	1.0821(4)	-0.0614(3)	0.1591(2)	0.023(1)
C(18)	1.2991(4)	-0.0142(3)	0.20065(19)	0.023(1)
C(19)	1.1776(3)	0.1457(3)	0.2373(2)	0.021(1)
C(20)	1.3726(4)	-0.2354(4)	0.0928(2)	0.031(1)
Cl(3)	1.27268(11)	0.09317(12)	-0.04943(6)	0.049(1)
O(2)	1.3559(4)	-0.2499(4)	-0.1247(2)	0.058(1)
C(21)	1.5238(5)	-0.3128(4)	-0.1540(3)	0.041(1)
H(1N)	0.458(5)	0.740(4)	0.468(3)	0.028(9)
H(2N)	1.263(4)	-0.021(4)	0.083(3)	0.022(8)
H(2)	0.19254	1.30405	0.81076	0.026
H(3)	-0.05856	1.48535	0.80241	0.028
H(4)	-0.17437	1.45723	0.68822	0.028
H(5)	-0.04196	1.24358	0.57980	0.025
H(14)	1.02072	0.28824	0.41530	0.025
H(15)	0.83386	0.51151	0.50488	0.024
H(12)	0.71955	0.33798	0.25510	0.024
H(16)	0.96843	0.16792	0.14635	0.026
H(16B)	0.85111	0.10441	0.22040	0.026

H(17)	1.07153	-0.13074	0.20843	0.027
H(17B)	1.05248	-0.09284	0.10691	0.027
H(18)	1.41091	-0.01486	0.17558	0.027
H(18B)	1.30000	-0.08084	0.25223	0.027
H(19)	1.20662	0.18183	0.28824	0.025
H(19B)	1.18290	0.21429	0.18706	0.025
H(20A)	1.34390	-0.27178	0.04259	0.046
H(20B)	1.36530	-0.29971	0.14436	0.046
H(20C)	1.48524	-0.24027	0.06950	0.046
H(2O)	1.32269	-0.15563	-0.11220	0.088
H(21A)	1.56379	-0.41329	-0.13084	0.061
H(21B)	1.56595	-0.24689	-0.13001	0.061
H(21C)	1.56229	-0.32331	-0.22214	0.061

Table S9. Anisotropic displacement parameters (Å^2) for ndm552_sq.
The anisotropic displacement factor exponent takes the form:
$-2\pi^2[h^2a^{*2}U_{11} + ... + 2hka^*b^*U_{12}]$

	U_{11}	U_{22}	U_{33}	U_{23}	U_{13}	U_{12}
Cl(1)	0.0211(3)	0.0241(3)	0.0214(3)	-0.0007(2)	-0.0094(2)	-0.0062(3)
Cl(2)	0.0229(3)	0.0254(3)	0.0301(4)	-0.0028(3)	-0.0141(3)	-0.0027(3)
S(1)	0.0196(3)	0.0198(3)	0.0196(3)	0.0007(2)	-0.0075(2)	-0.0063(3)
O(1)	0.0237(10)	0.0251(10)	0.0255(10)	-0.0027(8)	-0.0113(8)	-0.0023(8)
N(1)	0.0179(11)	0.0175(11)	0.0197(11)	0.0018(9)	-0.0071(9)	-0.0043(9)
N(2)	0.0183(11)	0.0178(11)	0.0208(11)	-0.0002(9)	-0.0051(9)	-0.0070(9)
N(3)	0.0233(12)	0.0200(11)	0.0151(11)	0.0034(9)	-0.0043(9)	-0.0079(10)
C(1)	0.0193(13)	0.0192(13)	0.0190(13)	0.0046(10)	-0.0045(10)	-0.0090(11)
C(2)	0.0271(14)	0.0214(13)	0.0198(13)	0.0027(10)	-0.0059(11)	-0.0127(12)
C(3)	0.0265(14)	0.0180(13)	0.0214(13)	-0.0002(10)	-0.0017(11)	-0.0086(11)
C(4)	0.0198(13)	0.0207(13)	0.0255(14)	0.0052(11)	-0.0040(11)	-0.0051(11)
C(5)	0.0212(13)	0.0216(13)	0.0190(13)	0.0045(10)	-0.0063(10)	-0.0088(11)
C(6)	0.0203(13)	0.0193(13)	0.0166(12)	0.0045(10)	-0.0036(10)	-0.0103(11)
C(7)	0.0208(13)	0.0211(13)	0.0168(12)	0.0034(10)	-0.0046(10)	-0.0103(11)
C(8)	0.0208(13)	0.0204(13)	0.0161(12)	0.0027(10)	-0.0050(10)	-0.0103(11)
C(9)	0.0195(13)	0.0181(12)	0.0185(12)	0.0038(10)	-0.0046(10)	-0.0077(11)
C(10)	0.0198(13)	0.0155(12)	0.0174(12)	0.0033(10)	-0.0031(10)	-0.0068(10)
C(11)	0.0179(12)	0.0182(13)	0.0213(13)	0.0057(10)	-0.0068(10)	-0.0078(10)
C(13)	0.0203(13)	0.0155(12)	0.0199(13)	0.0042(10)	-0.0034(10)	-0.0075(10)
C(14)	0.0208(13)	0.0204(13)	0.0201(13)	0.0038(10)	-0.0075(11)	-0.0060(11)
C(15)	0.0223(13)	0.0196(13)	0.0173(12)	0.0021(10)	-0.0063(10)	-0.0069(11)
C(12)	0.0222(13)	0.0202(13)	0.0186(13)	0.0026(10)	-0.0068(10)	-0.0101(11)
C(16)	0.0207(13)	0.0219(14)	0.0238(14)	0.0009(11)	-0.0074(11)	-0.0087(11)
C(17)	0.0241(14)	0.0216(14)	0.0221(13)	0.0006(11)	-0.0042(11)	-0.0113(11)
C(18)	0.0216(13)	0.0244(14)	0.0195(13)	-0.0010(11)	-0.0074(11)	-0.0057(11)
C(19)	0.0185(13)	0.0203(13)	0.0231(13)	-0.0006(10)	-0.0049(11)	-0.0066(11)
C(20)	0.0312(16)	0.0244(15)	0.0282(16)	-0.0043(12)	-0.0047(13)	-0.0050(13)
Cl(3)	0.0379(5)	0.0552(6)	0.0317(4)	0.0240(4)	-0.0026(3)	-0.0009(4)
O(2)	0.0541(18)	0.065(2)	0.0597(19)	0.0036(16)	-0.0209(15)	-0.0244(16)
C(21)	0.046(2)	0.045(2)	0.0326(18)	-0.0011(15)	-0.0057(15)	-0.0240(17)

Table S10. Bond lengths [Å] for ndm552_sq.

atom-atom	distance	atom-atom	distance
Cl(1)-C(7)	1.727(3)	Cl(2)-C(11)	1.746(3)
S(1)-C(1)	1.735(3)	S(1)-C(8)	1.745(3)
O(1)-C(9)	1.225(3)	N(1)-C(9)	1.362(4)
N(1)-C(10)	1.409(3)	N(1)-H(1N)	0.84(4)
N(2)-C(13)	1.418(3)	N(2)-C(16)	1.464(3)
N(2)-C(19)	1.472(3)	N(3)-C(20)	1.490(4)
N(3)-C(18)	1.491(3)	N(3)-C(17)	1.494(4)
N(3)-H(2N)	0.82(4)	C(1)-C(2)	1.399(4)
C(1)-C(6)	1.404(4)	C(2)-C(3)	1.374(4)
C(2)-H(2)	0.9500	C(3)-C(4)	1.403(4)
C(3)-H(3)	0.9500	C(4)-C(5)	1.383(4)
C(4)-H(4)	0.9500	C(5)-C(6)	1.409(4)
C(5)-H(5)	0.9500	C(6)-C(7)	1.434(4)
C(7)-C(8)	1.361(4)	C(8)-C(9)	1.490(4)
C(10)-C(11)	1.394(4)	C(10)-C(15)	1.399(4)
C(11)-C(12)	1.382(4)	C(13)-C(12)	1.395(4)
C(13)-C(14)	1.403(4)	C(14)-C(15)	1.382(4)
C(14)-H(14)	0.9500	C(15)-H(15)	0.9500
C(12)-H(12)	0.9500	C(16)-C(17)	1.509(4)
C(16)-H(16)	0.9900	C(16)-H(16B)	0.9900
C(17)-H(17)	0.9900	C(17)-H(17B)	0.9900
C(18)-C(19)	1.507(4)	C(18)-H(18)	0.9900
C(18)-H(18B)	0.9900	C(19)-H(19)	0.9900
C(19)-H(19B)	0.9900	C(20)-H(20A)	0.9800
C(20)-H(20B)	0.9800	C(20)-H(20C)	0.9800
O(2)-C(21)	1.363(5)	O(2)-H(2O)	0.8400
C(21)-H(21A)	0.9800	C(21)-H(21B)	0.9800
C(21)-H(21C)	0.9800		

Symmetry transformations used to generate equivalent atoms:

Table S11. Bond angles [°] for ndm552_sq.

atom-atom-atom	angle	atom-atom-atom	angle
C(1)-S(1)-C(8)	91.81(13)	C(9)-N(1)-C(10)	128.4(2)
C(9)-N(1)-H(1N)	116(3)	C(10)-N(1)-H(1N)	116(3)
C(13)-N(2)-C(16)	115.9(2)	C(13)-N(2)-C(19)	114.3(2)
C(16)-N(2)-C(19)	109.8(2)	C(20)-N(3)-C(18)	111.0(2)
C(20)-N(3)-C(17)	111.3(2)	C(18)-N(3)-C(17)	110.5(2)
C(20)-N(3)-H(2N)	110(2)	C(18)-N(3)-H(2N)	107(2)
C(17)-N(3)-H(2N)	107(2)	C(2)-C(1)-C(6)	121.7(3)
C(2)-C(1)-S(1)	126.7(2)	C(6)-C(1)-S(1)	111.6(2)
C(3)-C(2)-C(1)	117.7(3)	C(3)-C(2)-H(2)	121.2
C(1)-C(2)-H(2)	121.2	C(2)-C(3)-C(4)	121.8(3)
C(2)-C(3)-H(3)	119.1	C(4)-C(3)-H(3)	119.1
C(5)-C(4)-C(3)	120.8(3)	C(5)-C(4)-H(4)	119.6
C(3)-C(4)-H(4)	119.6	C(4)-C(5)-C(6)	118.6(3)
C(4)-C(5)-H(5)	120.7	C(6)-C(5)-H(5)	120.7
C(1)-C(6)-C(5)	119.5(3)	C(1)-C(6)-C(7)	111.0(2)
C(5)-C(6)-C(7)	129.5(3)	C(8)-C(7)-C(6)	114.4(2)
C(8)-C(7)-Cl(1)	125.9(2)	C(6)-C(7)-Cl(1)	119.7(2)
C(7)-C(8)-C(9)	135.1(3)	C(7)-C(8)-S(1)	111.2(2)
C(9)-C(8)-S(1)	113.71(19)	O(1)-C(9)-N(1)	124.8(3)
O(1)-C(9)-C(8)	119.4(2)	N(1)-C(9)-C(8)	115.9(2)
C(11)-C(10)-C(15)	116.7(2)	C(11)-C(10)-N(1)	118.8(2)
C(15)-C(10)-N(1)	124.5(2)	C(12)-C(11)-C(10)	123.4(2)
C(12)-C(11)-Cl(2)	117.4(2)	C(10)-C(11)-Cl(2)	119.2(2)
C(12)-C(13)-C(14)	117.8(2)	C(12)-C(13)-N(2)	122.3(2)
C(14)-C(13)-N(2)	119.9(2)	C(15)-C(14)-C(13)	121.9(3)
C(15)-C(14)-H(14)	119.0	C(13)-C(14)-H(14)	119.0
C(14)-C(15)-C(10)	120.7(3)	C(14)-C(15)-H(15)	119.6
C(10)-C(15)-H(15)	119.6	C(11)-C(12)-C(13)	119.5(2)
C(11)-C(12)-H(12)	120.2	C(13)-C(12)-H(12)	120.2
N(2)-C(16)-C(17)	110.5(2)	N(2)-C(16)-H(16)	109.6
C(17)-C(16)-H(16)	109.6	N(2)-C(16)-H(16B)	109.6
C(17)-C(16)-H(16B)	109.6	H(16)-C(16)-H(16B)	108.1
N(3)-C(17)-C(16)	111.3(2)	N(3)-C(17)-H(17)	109.4
C(16)-C(17)-H(17)	109.4	N(3)-C(17)-H(17B)	109.4
C(16)-C(17)-H(17B)	109.4	H(17)-C(17)-H(17B)	108.0
N(3)-C(18)-C(19)	111.3(2)	N(3)-C(18)-H(18)	109.4
C(19)-C(18)-H(18)	109.4	N(3)-C(18)-H(18B)	109.4
C(19)-C(18)-H(18B)	109.4	H(18)-C(18)-H(18B)	108.0
N(2)-C(19)-C(18)	110.5(2)	N(2)-C(19)-H(19)	109.6
C(18)-C(19)-H(19)	109.6	N(2)-C(19)-H(19B)	109.6
C(18)-C(19)-H(19B)	109.6	H(19)-C(19)-H(19B)	108.1
N(3)-C(20)-H(20A)	109.5	N(3)-C(20)-H(20B)	109.5

H(20A)-C(20)-H(20B)	109.5	N(3)-C(20)-H(20C)	109.5
H(20A)-C(20)-H(20C)	109.5	H(20B)-C(20)-H(20C)	109.5
C(21)-O(2)-H(2O)	109.5	O(2)-C(21)-H(21A)	109.5
O(2)-C(21)-H(21B)	109.5	H(21A)-C(21)-H(21B)	109.5
O(2)-C(21)-H(21C)	109.5	H(21A)-C(21)-H(21C)	109.5
H(21B)-C(21)-H(21C)	109.5		

Symmetry transformations used to generate equivalent atoms:

Table S12. Torsion angles [°] for ndm552_sq.

atom-atom-atom-atom	angle	atom-atom-atom-atom	angle
C(8)-S(1)-C(1)-C(2)	179.8(3)	C(8)-S(1)-C(1)-C(6)	0.3(2)
C(6)-C(1)-C(2)-C(3)	-0.3(4)	S(1)-C(1)-C(2)-C(3)	-179.7(2)
C(1)-C(2)-C(3)-C(4)	0.1(4)	C(2)-C(3)-C(4)-C(5)	0.4(4)
C(3)-C(4)-C(5)-C(6)	-0.7(4)	C(2)-C(1)-C(6)-C(5)	-0.1(4)
S(1)-C(1)-C(6)-C(5)	179.5(2)	C(2)-C(1)-C(6)-C(7)	-179.9(2)
S(1)-C(1)-C(6)-C(7)	-0.4(3)	C(4)-C(5)-C(6)-C(1)	0.5(4)
C(4)-C(5)-C(6)-C(7)	-179.7(3)	C(1)-C(6)-C(7)-C(8)	0.2(3)
C(5)-C(6)-C(7)-C(8)	-179.6(3)	C(1)-C(6)-C(7)-Cl(1)	-179.58(19)
C(5)-C(6)-C(7)-Cl(1)	0.6(4)	C(6)-C(7)-C(8)-C(9)	-179.9(3)
Cl(1)-C(7)-C(8)-C(9)	-0.1(5)	C(6)-C(7)-C(8)-S(1)	0.0(3)
Cl(1)-C(7)-C(8)-S(1)	179.81(16)	C(1)-S(1)-C(8)-C(7)	-0.2(2)
C(1)-S(1)-C(8)-C(9)	179.7(2)	C(10)-N(1)-C(9)-O(1)	-1.2(5)
C(10)-N(1)-C(9)-C(8)	178.8(2)	C(7)-C(8)-C(9)-O(1)	177.7(3)
S(1)-C(8)-C(9)-O(1)	-2.2(3)	C(7)-C(8)-C(9)-N(1)	-2.2(5)
S(1)-C(8)-C(9)-N(1)	177.85(19)	C(9)-N(1)-C(10)-C(11)	-178.2(3)
C(9)-N(1)-C(10)-C(15)	1.0(4)	C(15)-C(10)-C(11)-C(12)	-0.5(4)
N(1)-C(10)-C(11)-C(12)	178.8(2)	C(15)-C(10)-C(11)-Cl(2)	-178.9(2)
N(1)-C(10)-C(11)-Cl(2)	0.4(3)	C(16)-N(2)-C(13)-C(12)	-1.0(4)
C(19)-N(2)-C(13)-C(12)	128.1(3)	C(16)-N(2)-C(13)-C(14)	177.9(2)
C(19)-N(2)-C(13)-C(14)	-53.0(3)	C(12)-C(13)-C(14)-C(15)	-0.6(4)
N(2)-C(13)-C(14)-C(15)	-179.5(2)	C(13)-C(14)-C(15)-C(10)	0.0(4)
C(11)-C(10)-C(15)-C(14)	0.5(4)	N(1)-C(10)-C(15)-C(14)	-178.7(3)
C(10)-C(11)-C(12)-C(13)	0.0(4)	Cl(2)-C(11)-C(12)-C(13)	178.4(2)
C(14)-C(13)-C(12)-C(11)	0.6(4)	N(2)-C(13)-C(12)-C(11)	179.5(2)
C(13)-N(2)-C(16)-C(17)	-168.8(2)	C(19)-N(2)-C(16)-C(17)	60.0(3)
C(20)-N(3)-C(17)-C(16)	177.4(2)	C(18)-N(3)-C(17)-C(16)	53.5(3)
N(2)-C(16)-C(17)-N(3)	-57.1(3)	C(20)-N(3)-C(18)-C(19)	-177.6(2)
C(17)-N(3)-C(18)-C(19)	-53.6(3)	C(13)-N(2)-C(19)-C(18)	167.8(2)
C(16)-N(2)-C(19)-C(18)	-60.1(3)	N(3)-C(18)-C(19)-N(2)	57.2(3)

Symmetry transformations used to generate equivalent atoms:

Table S13. Hydrogen bonds for ndm552_sq [Å and °].

D-H...A	d(D-H)	d(H...A)	d(D...A)	<(DHA)
N(1)-H(1N)...Cl(1)	0.84(4)	2.39(4)	3.131(2)	147(3)
N(1)-H(1N)...Cl(2)	0.84(4)	2.47(4)	2.939(2)	116(3)
N(3)-H(2N)...Cl(3)	0.82(4)	2.24(4)	3.049(2)	169(3)
O(2)-H(2O)...Cl(3)	0.84	2.41	3.227(4)	164.8

Symmetry transformations used to generate equivalent atoms:
